# Clearance of HIV infection by selective elimination of host cells capable of producing HIV

**DOI:** 10.1038/s41467-020-17753-w

**Published:** 2020-08-13

**Authors:** Min Li, Wei Liu, Tonya Bauch, Edward A. Graviss, Roberto C. Arduino, Jason T. Kimata, Min Chen, Jin Wang

**Affiliations:** 1 Immunobiology and Transplant Science Center, Houston Methodist Research Institute, Houston, TX 77030 USA; 2 Department of Pathology and Genomic Medicine, Houston Methodist Research Institute, Houston, TX 77030 USA; 3 Division of Infectious Diseases, Department of Internal Medicine, McGovern Medical School at The University of Texas Health Science Center, Houston, TX 77030 USA; 4 Department of Molecular Virology and Microbiology, Baylor College of Medicine, Houston, TX 77030 USA; 5 Department of Pathology and Immunology, Baylor College of Medicine, Houston, TX 77030 USA; 6 Department of Surgery, Weill Cornell Medical College, Cornell University, New York, NY 10065 USA

**Keywords:** Autophagy, Immune cell death, Viral reservoirs, HIV infections

## Abstract

The RNA genome of the human immunodeficiency virus (HIV) is reverse-transcribed into DNA and integrated into the host genome, resulting in latent infections that are difficult to clear. Here we show an approach to eradicate HIV infections by selective elimination of host cells harboring replication-competent HIV (SECH), which includes viral reactivation, induction of cell death, inhibition of autophagy and the blocking of new infections. Viral reactivation triggers cell death specifically in HIV-1-infected T cells, which is promoted by agents that induce apoptosis and inhibit autophagy. SECH treatments can clear HIV-1 in >50% mice reconstituted with a human immune system, as demonstrated by the lack of viral rebound after withdrawal of treatments, and by adoptive transfer of treated lymphocytes into uninfected humanized mice. Moreover, SECH clears HIV-1 in blood samples from HIV-1-infected patients. Our results suggest a strategy to eradicate HIV infections by selectively eliminating host cells capable of producing HIV.

## Abstract (web summary)

The latent human immunodeficiency virus (HIV) reservoir in patients poses a problem for HIV cure. Here, Li et al. show that a combination of compounds inducing viral reactivation and cell death, inhibiting autophagy and blocking new infections can eliminate HIV infection in 50% of humanized HIV infected mice and in blood samples from infected patients.

## Introduction

The acquired immunodeficiency syndrome (AIDS) is caused by HIV, which infects and depletes CD4^+^ T cells in the patients^1^. The RNA genome of HIV type 1 (HIV-1) is reverse-transcribed into DNA and integrated into the host genome, resulting in persistent infections that are difficult to eradicate^2^. Combination antiretroviral therapy (cART) targeting different stages of the HIV-1 replication cycle can effectively inhibit viral replication and prevent the onset of AIDS^3^. The seeding of refractory latent viral reservoir takes place rapidly in human HIV-1 patients and in Simian immunodeficiency virus (SIV)-infected rhesus monkeys^4,5^. A stable HIV-1 proviral reservoir persists during cART^6–8^. Continuous cART is necessary to prevent new virus production from the HIV-1 reservoir^9^. Interestingly, two leukemia patients infected with HIV-1 have been reported to be cured of the virus through regimens of total body irradiation or chemotherapy plus antibody-mediated depletion of lymphocytes, followed by transplantation of hematopoietic stem cells from homozygous CCR5Δ32 donors^10,11^. However, a cure strategy for HIV-1 infections that is practical for people living with HIV in the general population remains to be developed.

HIV-1 has evolved mechanisms to evade immune recognition. Inducing the expression of HIV-1 with latency reversal agents (LRAs) to display viral antigens may trigger immune responses against latently infected cells^12^. However, LRAs alone have not been shown to reduce or clear HIV infections^13^, suggesting the requirement for additional approaches. Using a long-acting antiviral therapy and adenovirus-mediated delivery of CRISPR-Cas9 to excise integrated HIV-1, it was shown that HIV-1 was cleared from two human cell-implanted mice^14^. It has been shown that only a small portion (<3%) of integrated HIV-1 is capable of producing the infectious virions, whereas most integrated proviruses are defective and pose no risks in producing the virus^15^. Thus, the majority of the infected cells, which are non-productively infected, do not need to be cleared to achieve a cure for HIV-1. So far, the task of finding the needle in a haystack to sort out and destroy the HIV-1 reservoir capable of producing the virus has been challenging.

Killing cells harboring intact HIV-1 proviruses would be ideal for clearing the HIV-1 reservoir. HIV-1 can trigger different cell death pathways in T cells^16–23^. Interestingly, productive HIV-1 infection induces caspase-dependent apoptosis in host cells, whereas abortive HIV-1 infection leads to pyroptosis^24^. We and others have found that cell types important for immunological memory, including memory B cells, and CD4^+^, and CD8^+^ memory T cells, depend on autophagy for their long-term survival^25–28^. Because CD4^+^ memory T cells are the major reservoir for latent HIV-1, we hypothesize that targeting autophagy would facilitate the elimination of latent HIV-1 infection. By a selective elimination of host cells harboring replication-competent HIV (SECH) approach with the combination of latency reversal, inhibition of autophagy and induction of apoptosis, we show that it is feasible to clear host cells harboring replication-competent HIV-1 in humanized mice in vivo, as well as in blood samples from HIV-1-infected patients in vitro.

## Results

### Inhibition of autophagy promotes host cell apoptosis

**Fig. 1:**
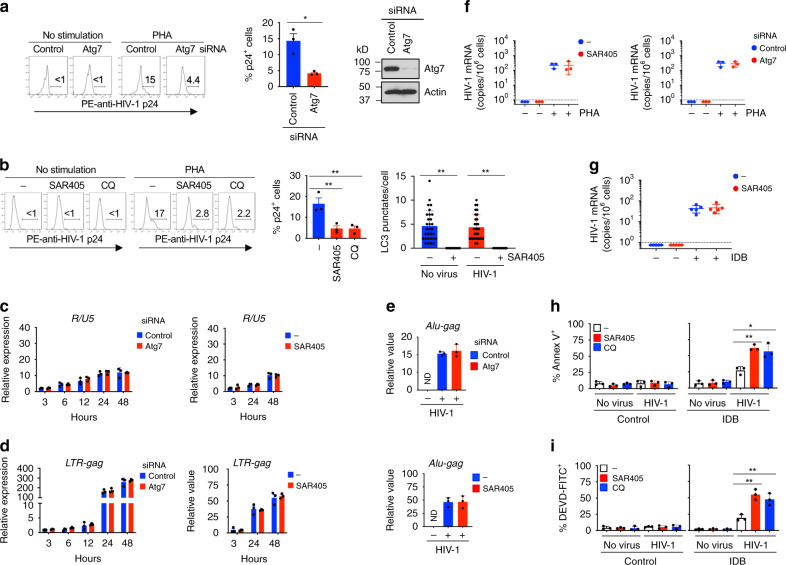
Regulation of host cell survival, but not HIV-1 reverse transcription and integration into the host genome by inhibition of autophagy. **a** CMT transfected with Atg7 siRNA were infected with HIV-1 (NL4-3, 0.1 MOI) and cultured for 4 d to establish latency. After latency reversal by PHA, p24^+^ cells (*n* = 3 biologically independent samples) and Atg7 expression (representative of two biologically independent experiments) were determined. *p = 0.011. **b** CMT latently infected with HIV-1 were stimulated with IDB with SAR405 (2 μm) or CQ (10 μm). p24 staining and the number of LC3 punctate per cells were analyzed. p24^+^ cells (*n* = 3 biologically independent experiments), *p* = 0.010 (SAR405) and 0.0098 (CQ); LC3 punctate/cell (*n* = 30 cells from two biologically independent experiments), *p* = 0.0001 (no virus) and 0.0001 (HIV-1). **c**, **d** Atg7 siRNA-transfected CMT were infected with HIV-1. Alternatively, CMT infected with HIV-1 were cultured with SAR405. Cells at different time after infection were collected (*n* = 3 biologically independent samples) for RT-PCR for *R/U5***c** and *LTR-gag***d**. **e***Alu-gag* PCR for genomic DNA from CMT as in **a**, CMT treated with SAR405 as in **b** or uninfected CMT. Data were normalized against β-globin. ND: not detectable. Data are presented as mean ± SD (*n* = 3 biologically independent samples). **f**, **g** CMT cultured as in **a**, **b** were reactivated with PHA for 24 h **f** (*n* = 3 biologically independent samples). PBMCs from ART-treated HIV-1-infected patients (*n* = 5 patients) were stimulated with IDB in the presence or absence of SAR405 for 24 h **g**. HIV-1 mRNA was determined by RT-PCR. Data are presented as mean ± SD. The dashed line indicates detection limit. **h**, **i** CMT latently infected with HIV-1 (NL4-3, 1 MOI) as in **a** were reactivated with 100 nm IDB for 24 h. Annexin V **h** or DEVD **i** staining were analyzed by flow cytometry (*n* = 3 biologically independent samples). SAR405 vs. control, *p* = 0.0012 **h**, 0.0024 **i**; CQ vs. control, *p* = 0.0164 **h**, 0.0088 **i**. Source data are provided as a Source Data file.

As autophagy is important for the protection of memory T cells, we tested whether inhibition of autophagy can help to reduce the HIV-1 reservoir in these cells. We used CD3^+^CD4^+^CD45RO^+^CCR7^+^ central memory T cells (CMT; Supplementary Fig. 1a) for HIV-1 infection and culture in the presence of CCL19 to establish HIV latent infection^29^. Four days after infection with CXCR4-tropic HIV-1 NL4-3^30^ at 0.1 multiplicity of infection (MOI), CMT showed no detectable expression of HIV-1 p24 protein (Fig. 1a, Supplementary Fig. 1b). Stimulation with phytohemagglutinin (PHA) led to latency reversal of HIV-1 as shown by the induction of p24 (Fig. 1a, Supplementary Fig. 1b). Latency reversal was also observed by the expression of HIV-1 mRNA after stimulation with PHA or ingenol-3,20-dibenzoate (IDB), a non-tumorigenic protein kinase c-ε activator^31^ (Supplementary Fig. 1c). Interestingly, inhibition of autophagy in T cells by silencing the expression of an essential autophagy gene, *Atg7*^32^, reduced the number of HIV-1 p24^+^ cells after PHA stimulation (Fig. 1a). Consistently, SAR405, an autophagy inhibitor that prevents autophagy initiation by suppressing VPS34^33^, decreased the number of HIV-1 p24-producing T cells after latency reversal (Fig. 1b, Supplementary Fig. 2a). Chloroquine (CQ), another autophagy inhibitor that blocks the progression of autophagolysomes^34^, also reduced the number of HIV-1 p24-producing T cells after latency reversal (Fig. 1b). We also established HIV latency in CMT infected with CCR5-utilizing HIV-1 AD8^35^ at 1 MOI, and induced latency reversal with IDB (Supplementary Fig. 1c). Induction of HIV-1 p24 expression by IDB-induced latency reversal was also inhibited by SAR405 (Supplementary Fig. 2b). These data suggest that inhibition of autophagy can reduce the numbers of cells capable of producing HIV-1.

Inhibition of autophagy may reduce HIV-1 p24^+^ cells after latency reversal by affecting HIV-1 reverse transcription, integration of viral DNA into the host genome, viral reactivation or host cell survival. To distinguish between these possibilities, we measured early and late products of reverse transcription by *R/U5* and *LTR-gag* reverse transcription polymerase chain reaction (RT-PCR), respectively^36^. We observed that the production of early and late HIV-1 transcripts, which indicates the level of reverse transcription, was not affected by silencing of Atg7 or treatment with SAR405 (Fig. 1c, d). We also found that inhibition of autophagy did not affect HIV-1 integration into the host genome by *Alu-gag* PCR^37^ (Fig. 1e). Induction of HIV-1 mRNA expression from latently infected cells was also unaffected by inhibition of autophagy (Fig. 1f). Moreover, induction of HIV-1 mRNA in latently infected peripheral blood mononuclear cells (PBMCs) from ART-treated HIV-1 patients was not changed by SAR405 (Fig. 1g), indicating that autophagy is not required for the reactivation of latently infected HIV-1. Together, these data suggest that inhibition of autophagy does not have a direct effect on reverse transcription, integration, and reactivation of latent HIV-1.

Interestingly, we observed that latency reversal with IDB-induced cell death in HIV-1-infected CMT, as shown by annexin V staining (Fig. 1h), and increased caspase-3 activities measured by cleavage of DEVD (Fig. 1i). Moreover, IDB-induced cell death in HIV-1-infected CMT was promoted by autophagy inhibitors SAR405 and CQ (Fig. 1h, i). These data suggest that inhibition of autophagy promotes host cell death during latency reversal.

### Specific killing of host cells by HIV-1 reactivation

**Fig. 2:**
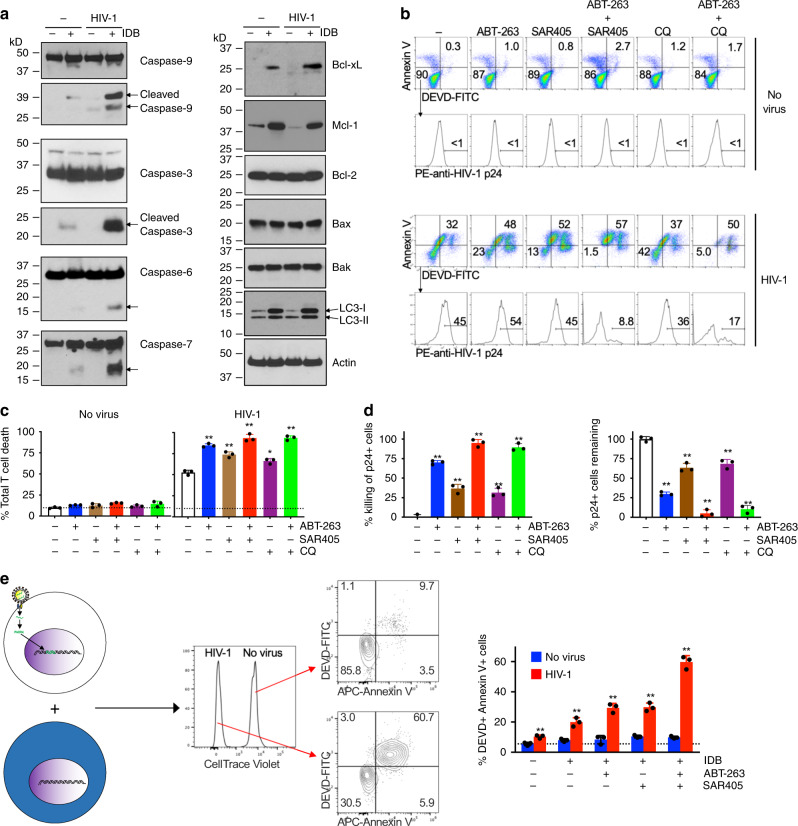
Induction of caspase activation and cell death in HIV-1-infected T cells. **a** CD4^+^ T cells from PBMCs with or without infection by HIV-1 (NL4-3, 1 MOI) were cultured for 4 days to establish latency, followed by stimulation with IDB for 24 h. Cell lysates were used for western blot (representative of two biologically independent experiments). Arrows indicate cleaved caspases. **b** CMT with or without infection by HIV-1 (NL4-3, 1 MOI) were cultured for 4 days to establish latency. The cells were stimulated with 0.1 μm IDB. ABT-263 (0.2 μm) and SAR405 (2 μm) and chloroquine (CQ, 10 μm) were added as indicated. The cells were cultured for 48 h, followed by incubation with DEVD-FITC, staining with APC-Annexin V, and intracellular staining with PE-anti-HIV p24. **c** Total cell death for cells treated in **b** was calculated (*n* = 3 biologically independent samples). Data are presented as mean ± SD. *p* values for control vs. five treatment groups in sequence: 0.0001, 0.0011, 0.0002, 0.0049, and 0.0001 (one-way ANOVA with unpaired two-tailed *t* test). **d** The combinations of ABT-263 and SAR405 or CQ in the killing of IDB-stimulated HIV-1-infected T cells in **b** was calculated. The remaining viable HIV-1 p24^+^ cells (negative for staining by APC-annexin V and DEVD-FITC) in **b** were also calculated. Data are presented as mean ± SD (*n* = 3 biologically independent samples). *p* values for control vs. four treatment groups in sequence (for killing of p24^+^ cells and for p24^+^ cells remaining): 0.0001, 0.0006, 0.0001, 0.0011, and 0.0001 (one-way ANOVA with unpaired two-tailed *t* test). **e** T cells latently infected with HIV-1 as in Fig. 1a were mixed with CellTrace violet-labeled uninfected T cells, and cultured with IDB, SAR405, and ABT-263 as in **b**, in the presence of 0.2 μm BMS-626529 for 48 h. The cells were stained with DEVD-FITC and APC-Annexin V, followed by flow cytometry analysis. Data are presented as mean ± SD and are representative of five independent experiments. *p* values for control vs. four treatment groups (HIV-1-infected samples) in sequence: 0.0059, 0.0005, 0.0004, and 0.0001 (one-way ANOVA with unpaired two-tailed *t* test). Source data are provided as a Source Data file.

We found that IDB-mediated latency reversal induced the activation of caspase-9, caspase-3, caspase-6, and caspase-7 in HIV-1-infected T cells, as shown by the appearance of active processed forms of these caspases (Fig. 2a). This is consistent with the possibility that latency reversal by IDB induces cell death in HIV-1-infected host cells (Fig. 1h, i). Treatment with IDB did not change the expression of antiapoptotic Bcl-2, but increased the expression of antiapoptotic Bcl-xL and Mcl-1 in CD4^+^ T cells with or without HIV-1 infections (Fig. 2a). The increase in Bcl-xL expression in HIV-1-infected cells was greater than in uninfected controls (Fig. 2a). This indicates that HIV replication may synergize with IDB in inducing antiapoptotic Bcl-xL, reminiscent of the roles for viral components in the regulation of Bcl-xL^38^. Moreover, IDB also induced the expression of LC3 in T cell with or without HIV-1 infections (Fig. 2a), indicating that IDB promotes autophagy in T cells. Although IDB can induce virus production in T cells harboring latent HIV-1 to trigger apoptosis, the upregulation of antiapoptotic molecules and autophagy by IDB would counteract apoptosis signaling. This may explain in part why the use of LRAs alone is not sufficient to clear HIV-1-infected cells. Nevertheless, the induction of antiapoptotic molecules and autophagy by IDB may have the advantage by conferring resistance of uninfected T cells to the induction of cell death.

### Killing of host cells by targeting apoptosis and autophagy

Virus reactivation by IDB-induced cell death in HIV-1-infected T cells as shown by staining with Annexin V and cleavage of DEVD, whereas uninfected cells were relatively resistant (Fig. 2b, c). Inhibition of autophagy with SAR405 increased IDB-induced killing of T cells latently infected by HIV-1 (Fig. 2b, c). Because latency reversal by IDB also showed the unintended effect of increasing antiapoptotic molecules (Fig. 2a), targeting antiapoptotic molecules in addition to reactivating the virus is potentially advantageous in promoting the killing of HIV-infected cells. Indeed, an inhibitor of Bcl-2 and Bcl-xL, ABT-263^39^, increased IDB-mediated cell death in latently infected T cells, as shown by staining with Annexin V and DEVD-FITC (Fig. 2b, c). ABT-263 significantly increased the loss of viable HIV-1 p24^+^ cells in IDB-stimulated HIV-1-infected cells (Fig. 2d). This suggests that counteracting antiapoptotic molecules and inhibiting autophagy can promote the killing of HIV-1-infected T cells induced by latency reversal.

To eradicate HIV infections, all T cells that are capable of producing infectious HIV-1 need to be cleared. We found that combining SAR405 with ABT-263 and IDB further increased cell death in HIV-infected T cells (Fig. 2b, c). Moreover, over 95% of HIV-1-p24^+^ cells in HIV-1-infected T cells could be killed within 2 days in vitro (Fig. 2d), whereas uninfected T cells were relatively resistant (Fig. 2b, c). Use of CQ instead of SAR405 also achieved similar results (Fig. 2b–d). These results suggest that latency reversal in combination with inhibition of autophagy and induction of apoptosis could efficiently kill HIV-1-infected T cells.

HIV-1 can establish latency in both resting and activated T cells^40^. In addition to memory T cells, certain levels of autophagy are present in T cells at all developmental stages^41^. We therefore examined whether inhibition of autophagy might affect the survival of HIV-1-infected CD4^+^ T cells with different differentiation and activation status in addition to CMT, including CD3^+^CD4^+^CD45RO^+^CCR7^−^ effector memory T cells (EMT), CD3^+^CD4^+^CD45RO^−^CCR7^+^ naive T cells (Supplementary Fig. 1a), as well as CD4^+^ T cells activated with PHA and IL-2. We found that the combination of IDB-induced latency reversal with inhibition of autophagy and promotion of apoptosis could kill a majority of these T cells infected by HIV-1 but not uninfected controls (Supplementary Fig. 3a–d). These results support the conclusion that the killing of HIV-1-infected T cells by latency reversal in combination with inhibition of autophagy and induction of apoptosis is selective.

### Specific killing of HIV-1-infected cells but not bystanders

To further determine the selectivity in the killing of HIV-1-infected cells by latency reversal with inhibition of autophagy and promotion of apoptosis, we labeled uninfected T cells with CellTrace Violet and mixed them with T cells latently infected by HIV-1 (Fig. 2e). An attachment inhibitor that targets HIV-1 gp120 and blocks its binding to CD4^+^ T cells, BMS-626529^42^, was added to prevent new rounds of infection. Treatments with IDB, ABT-263, and SAR4505 alone or together killed HIV-1-infected cells, whereas uninfected cells were resistant (Fig. 2e). These results suggest that IDB-induced latency reversal, in combination with the inhibition of autophagy and promotion of apoptosis, leads to selective killing of HIV-1-infected cells but not the uninfected bystanders.

Virus reactivation by IDB potentially confers the specificity in the killing of HIV-1-infected cells by selectively inducing caspase activation and apoptosis (Fig. 2a). The unintended effect of up regulating antiapoptotic molecules and autophagy by IDB may be mitigated by using ABT-263 and SAR405. Inhibition of autophagy could remove an important protective mechanism for cell survival, thereby unleashing the cell death pathways in HIV-1-infected T cells triggered by IDB and by proapoptotic ABT-263 (Fig. 2b, c; Supplementary Fig. 3). IDB-induced upregulation of antiapoptotic molecules and the induction of autophagy in cells not infected by HIV-1 may protect these cells against cell death (Fig. 2a), thereby increasing the specificity in the killing of HIV-1-infected cells.

### Clearance of HIV-1 infections in humanized mice by SECH

**Fig. 3:**
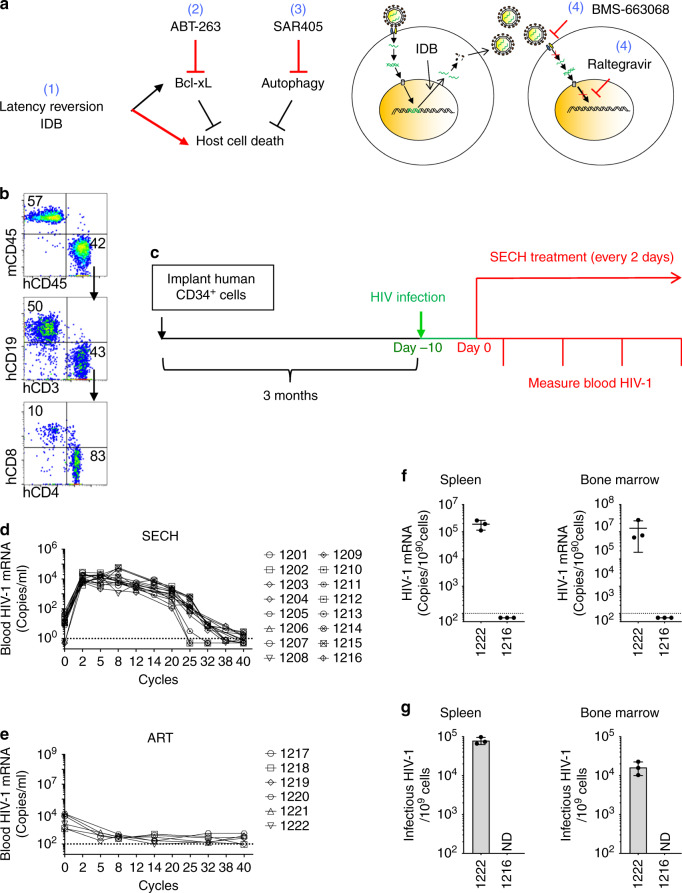
Treatment of HIV-1 infections in Hu-HSC mice by SECH. **a** The SECH regimen includes: (1) latency reversion; (2) induction of cell death; (3) inhibition of autophagy; and (4) blocking of new infections with inhibitors for HIV-1 attachment and integration. **b** An example of flow cytometry analyses of human cells in the peripheral blood of NSG-SGM3 mice 3 months after reconstitution with human CD34^+^ stem cells. Cell negative for mouse CD45 (mCD45) and positive for human CD45 (hCD45) were gated to analyze CD19^+^ human B cells, CD3^+^CD4^+^, and CD3^+^CD8^+^ human T cells. **c** Three months after reconstitution with human CD34^+^ stem cells, one set of HIV-1-infected Hu-HSC mice (Supplementary Table 1) were infected with HIV-1 (AD8 strain, 1000 pfu/mouse) intraperitoneally. Ten days after HIV-1 infections, the mice were used for treatments by ART or SECH. **d**, **e** RNA from the whole blood (including plasma and cells) was extracted to measure HIV-1 mRNA in mice treated by SECH **e** or ART **f** for 40 cycles. The dash line indicates detection limit. **f** HIV-1 mRNA in the spleen and bone marrow of mice treated by SECH or ART was determined by RT-PCR. Data are presented as mean ± SD (*n* = 3 technical replicates). ND, not detectable. **g** Infectious HIV-1 in the spleen and bone marrow of mice treated by SECH or ART was measured by TZA assays. Data are presented as mean ± SD (*n* = 3 technical replicates). Source data are provided as a Source Data file.

To determine whether it was possible to clear HIV-1 infection in vivo by killing HIV-1-infected cells, we used a humanized mouse model containing a reconstituted human immune system that had been established for HIV-1 infection and cure studies^43^. We designed a SECH approach for selective elimination of host cells capable of producing HIV, including the use of IDB for latency reversal, in combination with agents that induce apoptosis and inhibit autophagy to eliminate HIV-1-infected cells in humanized mice (Fig. 3a). However, IDB is expected to induce the production of new HIV-1 virions that can infect other cells. We included antiretroviral drugs that aim to inhibit the HIV-1 fusion and integration (Fig. 3a). We used a prodrug for attachment inhibitor BMS-626529, BMS-663068, with increased solubility and can be converted to the active and cell permeable BMS-626529 by alkaline phosphatase in the intestine^44^, as well as raltegravir, an integrase strand transfer inhibitor that prevents viral integration^45^. Other drugs commonly used in cART, such as reverse transcriptase inhibitors and protease inhibitors^3^, were not included. This would thus permit viral production to induce cell death signaling in infected cells while preventing virus spread, thereby protecting neighboring healthy cells from infection by newly produced HIV-1.

To generate mice implanted with human CD34^+^ hematopoietic stem cells (Hu-HSC mice), we used immunodeficient NSG-SGM3 mice with transgenic expression of human IL-3, granulocyte macrophage-colony stimulating factor (GM-CSF), and stem cell factor that support the stable engraftment of a variety of cell types in the immune system, including both myeloid and lymphoid lineages^46–48^. After engraftment of human CD34^+^ stem cells, mice were efficiently reconstituted with human CD4^+^ and CD8^+^ T cells, B cells, NK cells, dendritic cells, and macrophages (Fig. 3b; Supplementary Fig. 4a; Supplementary Table 1). Hu-HSC mice used for this set of experiment were reconstituted with an average of 33% human CD45^+^ cells (Supplementary Table 1). Among these human CD45^+^ cells, 40.8% were CD4^+^ T cells (Supplementary Table 1). These mice were then infected with HIV-1 (Fig. 3c). Recent data suggest that the establishment of refractory viral reservoirs takes place rapidly in human HIV-1 patients and in SIV-infected rhesus monkeys^4,5^. We examined some Hu-HSC mice 10 days after HIV-1 infection. Stimulation of splenocytes with PHA induced the expression of HIV-1 mRNA and production of Gag p24 in CD4^+^ T cells (Supplementary Fig. 4b, c). Although HIV-1 protein expression could still be detected in some T cells before the start of ART, these data suggest that latent HIV-1 infection has been established in human CD4^+^ T cells in these Hu-HSC mice.

We then determined the effects of SECH treatments in these mice in vivo. SECH treatments contained IDB (2.5 mg/kg b.w.), ABT-263 (50 mg/kg b.w.), and SAR405 (50 mg/kg b.w.) formulated in a solvent mixture 10% ethanol, 30% polyethylene glycol 400, and 60% Phosal 50 PG (EPP) for delivery into mice by oral gavage. Pilot experiments with administration of these doses of drugs to wild type C57BL/7 mice or in NSG-SGM3-derived HSC-Hu mice daily for 2 weeks showed no loss of body weight or other adverse effects on mice. SECH treatment was started at day 10 post infection once every 2 days as one cycle of treatments (Fig. 3c). Raltegravir and BMS-663068 (20 mg/kg b.w.) were included as the ART regimen daily. Mice in the control group received ART only daily.

We monitored HIV-1 mRNA in the mouse peripheral blood by RT-PCR. The purpose of ART included in the SECH protocol was to prevent the spread of HIV to uninfected cells, but not to decrease the production of new HIV-1 from infected cells induced by virus reactivation (Fig. 3a). We observed a burst of new HIV-1 production induced by virus reactivation in the SECH group (Fig. 3d). A decline in HIV-1 mRNA detected in the blood would suggest a reduction in the HIV-producing cell pool. Indeed, we found that HIV-1 in the peripheral blood decreased after 25–32 cycles of SECH treatments (Fig. 3d). Between 32 and 40 cycles of SECH treatments, most mice treated by SECH showed either reduced or undetectable HIV-1 in the peripheral blood (Fig. 3d). As expected, mice in the ART-treated group showed low or undetectable HIV-1 throughout the treatments (Fig. 3d).

### Determination of HIV-1 clearance in Hu-HSC mice

To determine whether HIV-1-producing cells were cleared in Hu-HSC after 40 cycles of SECH treatments, we examined HIV-1 in the spleen and bone marrow from mouse 1216 treated by SECH and mouse 1222 treated by ART. We could detect HIV-1 mRNA in the spleen and bone marrow of mouse 1222 but not mouse 1216 (Fig. 3f). TZM-bl cells stably expressing CD4, CCR5, CXCR4, and carrying a β-galactosidase gene under the control of HIV-1 long terminal repeat promoter have been used for more sensitive detection of replication-competent HIV-1 than traditional virus outgrowth assays or quantitative RT-PCR^49,50^. By this highly sensitive TZM-bl cell-based galactosidase reporter analysis (TZA) assay, we detected infectious HIV-1 in the spleen and bone marrow cells of mouse 1222 but not mouse 1216 (Fig. 3g). These results indicate that infectious HIV-1 is present in mouse 1222 treated by ART, but absent in mouse 1216 treated by SECH.

**Fig. 4:**
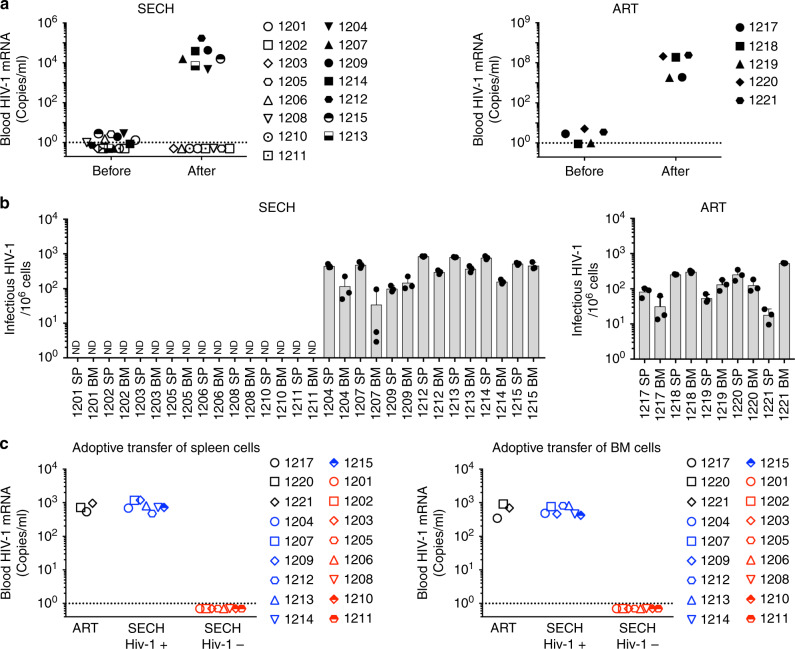
Clearance of HIV-1 infections in Hu-HSC mice by SECH. **a** HIV-1 viral RNA levels in the blood of SECH- or ART-treated Hu-HSC mice before and after 2 months of withdrawal of the treatments (ART, *n* = 5; SECH, *n* = 15). The dash line indicates detection limit. **b** Measuring infectious HIV-1 from spleen and bone marrow (BM) cells of HIV-1-infected Hu-HSC mice by TZA. Data are presented as mean ± SD (ART, *n* = 5 mice; SECH, *n* = 15 mice). Data are presented as mean ± SD. Each biological sample was measured in three technical replicates. ND, not detectable. **c** Virus outgrowth assay after adoptive transfer of spleen and BM cells from mice as in **c** into uninfected Hu-HSC recipient mice. (ART, *n* = 3 mice; SECH, *n* = 15 mice). Source data are provided as a Source Data file.

To further determine whether HIV-1 was cleared in Hu-HSC, SECH treatments were stopped after 40 cycles, followed by 2 months of withdrawal of treatments. We then measured HIV-1 in the peripheral blood of these mice. Eight mice were found to be negative for HIV-1 in the blood, whereas seven mice showed HIV-1 rebound after withdrawal of SECH (Fig. 4a). We found that those mice with no HIV-1 rebound in the blood also did not produce HIV-1 from spleen and bone marrow cells by the TZA assay (Fig. 4b). In contrast, all mice treated by ART showed virus rebound (Fig. 4a, b). In SECH-treated mice lacking detectable HIV-1 in the spleen and bone marrow, RT-PCR also showed no detectable HIV-1 in the lung, liver, and kidney (Supplementary Fig. 5a). Clearance of infectious HIV-1 is correlated with a decrease in HIV-1 DNA (Supplementary Fig. 5b). Lower levels of viral DNA are consistent with the clearance of productive HIV-1. The remaining viral DNA in mice with the clearance of productive infection likely represents non-productive HIV infection^51^. Consistent with this possibility, an HIV-1 patient treated by stem cell transplantation is free of the infectious virus but still contains residual HIV-1 DNA in the tissues^52^. These observations support the conclusion that HIV-1 cure can be achieved by clearing replication-competent HIV-1 without removing the non-productive virus. Intracellular staining of T cells also showed the lack of p24 production in mice free of HIV-1 (Supplementary Fig. 6a). In contrast, all mice with HIV-1 rebound showed HIV-1 production in spleen and bone marrow cells (Fig. 4b). All mice treated by ART also remained HIV-1^+^ (Fig. 4a, b). This indicates that HIV-1 was cleared from >50% of Hu-HSC mice treated by SECH. In contrast, ART did not clear HIV-1 although it could control active viral production. In parallel experiments, we observed that HIV-1 infections in Hu-HSC mice could be suppressed by ART (Supplementary Table 2, Supplementary Fig. 5c). Further treatments by SECH could clear HIV-1 in an apportion of these mice, as shown by lack of virus rebound after withdrawal of the treatments, and by TZA assays (Supplementary Fig. 5d–g). This suggests that SECH is potentially effective for treating HIV-1 infections with or without prior ART treatments.

### Validation of HIV clearance by an in vivo outgrowth assay

In an in vivo humanized mouse-based-virus outgrowth assay (hmVOA) through adoptive transfer of HIV-1-infected cells into humanized mice, the preformed lymphoid organs in the recipients provides highly sensitive detection of latent HIV-1 infections^14,53,54^. To further confirm virus clearance in the SECH-treated mice, we transferred spleen and bone marrow cells from these mice into uninfected Hu-HSC mice. Consistent with the in vitro TZA assay, HIV-1 was not detected by hmVOA in uninfected recipients after adoptive transfer of spleen or bone marrow cells from HIV-1-negative mice (Fig. 4c). In contrast, all Hu-HSC mice became HIV-1^+^ after receiving spleen or bone marrow cells from HIV-1^+^ mice (Fig. 4c). These data demonstrate that mice negative for HIV-1 by TZA assay were indeed free of virus by in vivo virus outgrowth assay in recipient Hu-HSC mice. These results suggest that the SECH treatments led to the elimination of HIV-1 infections in 8 out of 15 Hu-HSC mice as shown by the lack of virus rebound after withdrawal of SECH treatments, and the lack of infectious viruses by in vitro TZA assay and by in vivo hmVOA assay. We observed no significant loss of body weight in these mice after SECH or ART treatments (Supplementary Fig. 6b). We also observed no signs of inflammation or other histological changes in the brain, liver, lung, and kidney of these mice after SECH treatments (Supplementary Fig. 6c). SECH- and ART-treated mice showed overall comparable levels of human CD19^+^ B cells and CD4^+^ or CD8^+^ T cells (Supplementary Fig. 7a). In SECH-treated mice, there were increases in CD45RO^+^CCR7^−^ EMT cells and CD45RO^–^CCR7^−^ effector T cells, whereas CD45RO^+^CCR7^+^ CMT cells and CD19^+^CD27^+^ memory B cells were still present (Supplementary Fig. 7b). These results indicate that SECH is safe for treating HIV-1-infected Hu-HSC mice to clear HIV-1 infections.

### Synergy between JQ1 and IDB in HIV-1 clearance by SECH

Why HIV-1 is only cleared in a portion of the infected Hu-HSC mice is unclear. It is possible that improving HIV-1 reactivation induced by IDB could promote viral clearance. Bromodomain containing 4 is a negative regulator of transcription factor PTEF-b required for HIV-1 gene expression^55^. JQ1, an inhibitor for the BET family of bromodomains, can promote the reactivation of HIV-1^56^. Consistent previous observations^57^, we observed that using JQ1 and IDB together induced better reactivation of HIV-1 from latently infected T cells (Supplementary Fig. 8a). We therefore investigated whether including the epigenetic modifier JQ1 could increase the efficacy of SECH treatment.

**Fig. 5:**
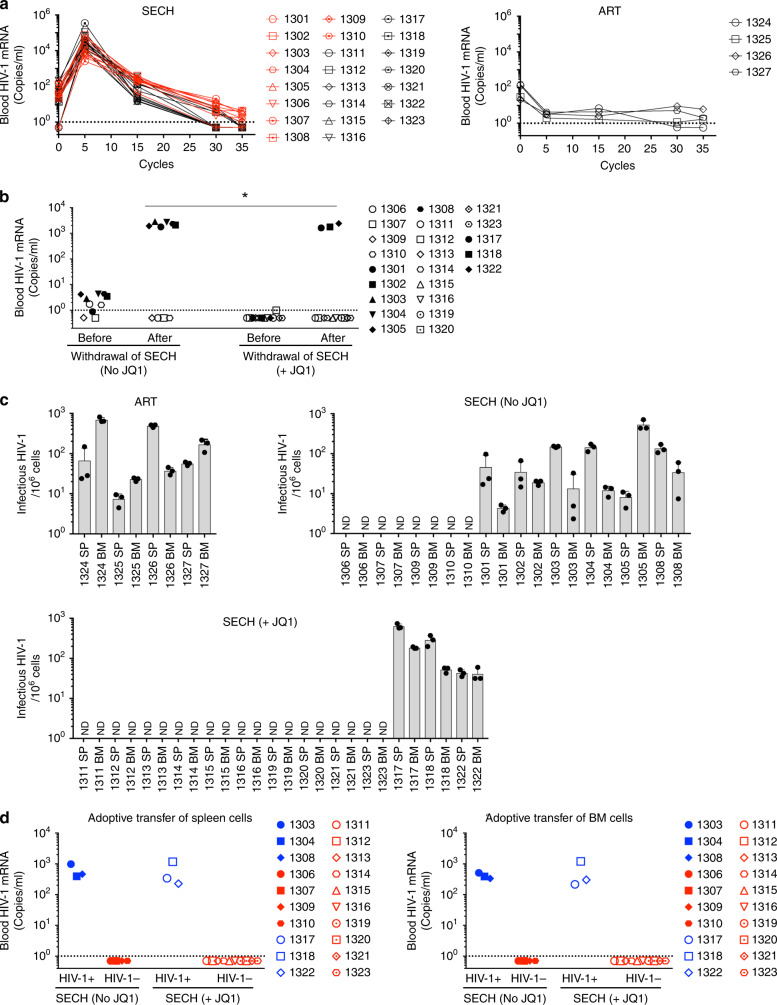
Improvement of SECH treatment by inclusion of JQ1. **a** Viral RNA levels in one set of HIV-1-infected Hu-HSC mice (Supplementary Table 3) orally treated by SECH with (black symbols) or without JQ1 (red symbols) for a total of 35 cycles (ART, *n* = 5 mice; SECH, *n* = 10 mice; SECH + JQ1, *n* = 13 mice). The dash line indicates detection limit. **b** Viral titer in the blood before and after withdrawal of the treatments. (SECH, *n* = 10 mice; SECH + JQ1, *n* = 13 mice). No JQ1 vs. JQ1, *p* = 0.0407 (Mann–Whitney test). **c** Measuring infectious HIV-1 from spleen and bone marrow cells of HIV-1-infected Hu-HSC mice by TZA. Biological samples from each mouse were measured in three technical replicates. Data are presented as mean ± SD (ART, *n* = 5 mice; SECH, *n* = 10 mice; SECH + JQ1, *n* = 13 mice). ND, not detectable. **d** hmVOA for spleen and bone marrow cells from mice in **c** (SECH, *n* = 7 mice; SECH + JQ1, *n* = 13 mice). Source data are provided as a Source Data file.

We compared SECH treatments with or without the inclusion of JQ1 in HIV-1-infected Hu-HSC mice. Hu-HSC mice were reconstituted with an average of 35.6% human CD45^+^ cells (Supplementary Table 3). Among human CD45^+^ cells, 46.6% were CD4^+^ T cells (Supplementary Table 3). After the initial burst of HIV-1 mRNA induced by virus reactivation by SECH, all mice showed either low or undetectable HIV-1 in the peripheral blood after 35 cycles of SECH treatments (Fig. 5a). After 2 months of withdrawal of treatments, 4 out of 10 mice in the SECH group without JQ1 showed no HIV-1 rebound (Fig. 5b). Interestingly, 10 out of 13 mice showed no virus rebound when JQ1 was included in the SECH treatments (Fig. 5b). No HIV-1 production was detected in the spleen and bone marrow cells from these HIV-1-negative mice by TZA assay (Fig. 5c). Moreover, HIV-1 was not detected by hmVOA assays after adoptive transfer of spleen and bone marrow cells from these HIV-1-negative mice into Hu-HSC recipients (Fig. 5d). In contrast, HIV-1^+^ mice after SECH treatments and all ART-treated mice produced HIV-1 by in vitro TZA and in vivo hmVOA assays (Fig. 5c, d). In addition,, we observed no significant differences in body weight, tissue sections and the total numbers of T or B cells in these mice after treatment by SECH or ART (Supplementary Fig. 8b–d). These results suggest that inclusion of JQ1 together with IDB for latency reversal can enhance the efficacy of HIV-1 clearance by SECH.

### Clearance of HIV-1 in PBMCs of HIV-1-infected patients

**Fig. 6:**
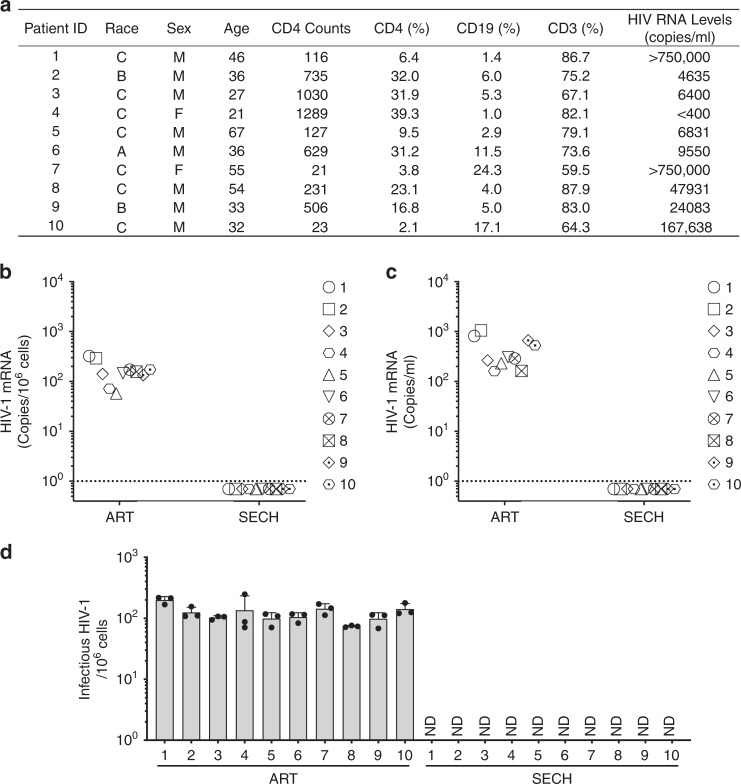
Clearance of HIV-1-infected cells from ART-naive HIV-1 patients by SECH treatments in vitro. **a** CD4 counts and HIV-1 load in ART-naive HIV-1-infected patients (*n* = 10) without previous antiretroviral treatment. A, Asian; B, Black; C, Caucasian. **b** PBMCs obtained from ART-naive HIV-1 patients (*n* = 10) were culture in vitro with agents for SECH or ART only for 2 days as on cycle, with a total of seven cycles as described in the “Method” section. Samples were also re-stimulated with IDB for detection of HIV-1 mRNA by RT-PCR. The dash line indicates detection limit. **c** PBMCs treated by in **b** were depleted of CD8^+^ T cells and 3 × 10^6^ cells were adoptively transferred into uninfected Hu-HSC mice for detection of HIV-1 by hmVOA. **d** The cells treated in **b** were used for TZA analyses. Ten biological samples were each measured in three technical replicates and presented as mean ± SD. Source data are provided as a Source Data file.

We next determined whether SECH could be used to clear HIV-1 infections in PBMCs from HIV-1-infected patients. We examined PBMCs from 10 ART-naive HIV-1 patients who had not received previous antiretroviral treatments. Five of these patients have relatively normal CD4^+^ T-cell counts (>500/μl blood), whereas four showed severely depletion of CD4^+^ T cells (<200/μl; Fig. 6a). PBMCs from each patient were separated into two fractions for treatments by either SECH or ART, with a 2-day culture as one cycle of treatments. After seven cycles of treatments, HIV-1 was undetectable in PBMCs treated by SECH, but remained detectable after treatment by ART (Fig. 6b). Consistent with RT-PCR analyses, SECH-treated PBMCs did not produce HIV-1 after adoptive transfer into Hu-HSC recipient mice, whereas the same patient samples treated by ART produced the virus in the recipients (Fig. 6c). TZA assays also showed the lack of infectious HIV-1 in cells treated by SECH (Fig. 6d). This suggests that the SECH treatments successfully eliminated HIV-1 in the blood samples of HIV-1-infected patients.

**Fig. 7:**
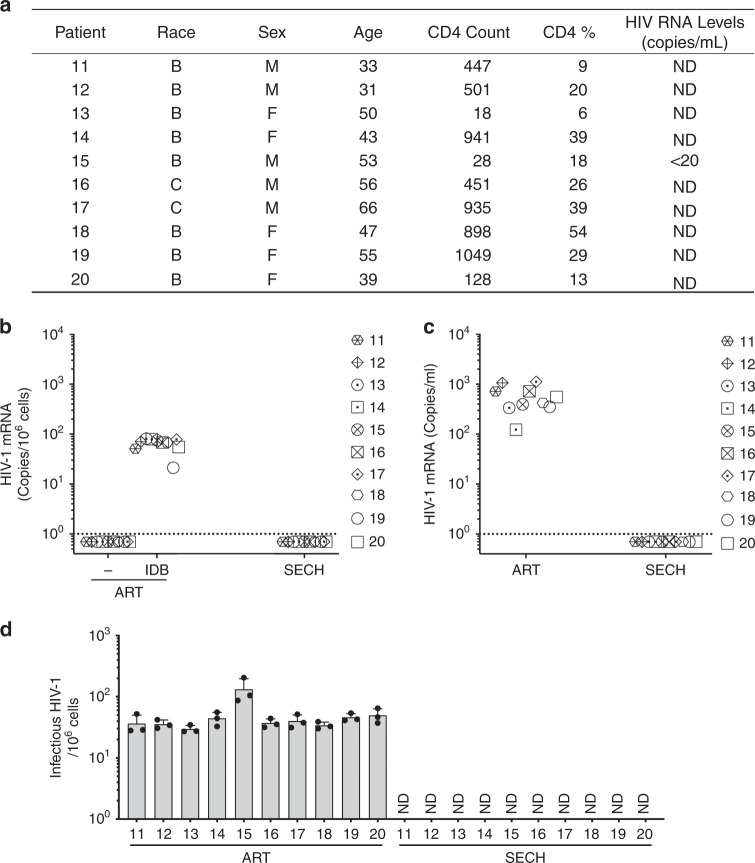
Clearance of HIV-1-infected cells from ART-experienced HIV-1 patients by SECH treatments in vitro. **a** CD4 counts and HIV-1 load in ART-experienced HIV-1-infected patients (*n* = 10). HIV viremia was successfully suppressed in all patients at the time of blood collection. Patients 18 and 19 had undergone ART treatments for 139 and 95 days, respectively. **b**–**d** PBMCs from ART-experienced HIV-1 patients (*n* = 10) were treated with SECH or ART and then used for detection of HIV-1 mRNA by RT-PCR **b** hmVOA **c** and TZA analyses **d** as in Fig. 6. Ten biological samples were each measured in three technical replicates and presented as mean ± SD **d**. Source data are provided as a Source Data file.

We also examined PBMCs from outpatients undergoing ART treatments at the Houston Methodist Hospital (Fig. 7a). Although PBMCs of these patients showed no active virus production, HIV-1 could be detected after stimulation with IDB (Fig. 7b, Supplementary Fig. 9a), suggesting that these patients harbored latent HIV-1 infections. After five cycles of treatments by SECH, we observed that HIV-1 was absent in PBMCs from these patients as shown by RT-PCR (Fig. 7b), indicating that SECH is effective for clearing HIV-1 in PBMCs from ART-treated patients. To confirm this finding, we next performed adoptive transfer of treated PBMCs into uninfected Hu-HSC mice for in vivo hmVOA assay. We found that SECH-treated PBMCs from these patients did not produce HIV-1, whereas all ART-treated samples were HIV-1^+^ by hmVOA (Fig. 7c). Consistently, TZA assays also showed that the cells treated by SECH did not produce infectious HIV-1 (Fig. 7d). SECH treatment without the inclusion of ART drugs failed to clear HIV-1 (Supplementary Fig. 9b), suggesting that blocking new viral infections is critical for the success of SECH. Similar to our earlier observations for in vitro cultures (Fig. 2e), the cell viabilities and total live cells of patient PBMCs were similar between untreated controls and samples treated by SECH or ART (Supplementary Fig. 9c). Interestingly, the levels of total T cells were comparable between samples treated by SECH and ART (Supplementary Fig. 9d). Memory B cells were comparable in samples treated by SECH and ART (Supplementary Fig. 9e). In SECH-treated samples, there were increases in CD45RO^+^CCR7^−^ EMT cells and CD45RO^−^CCR7^−^ effector T cells, while CD45RO^+^CCR7^+^ CMT cells were not significantly changed (Supplementary Fig. 9f). This indicates that SECH treatments killed infected T cells but not uninfected cells. SECH therefore can potentially be developed into an effective strategy to treat HIV-1 infections.

## Discussion

We show that it is feasible to clear HIV-1 infection by the SECH approach through selective elimination of host cells capable of producing the virus. This method combines agents that reactivate HIV-1, promote cell death, and inhibit autophagy, together with ART to prevent new infections. Continuous cycles of SECH treatments cleared HIV-1 infection in over 50% of Hu-HSC mice in vivo. HIV-1 clearance was confirmed by a lack of virus rebound after withdrawal of SECH treatments, in vitro TZA assays, and in vivo virus outgrowth assay by adoptive transfer of the spleen and bone marrow cells from treated mice into uninfected humanized mice. HIV-1 was cleared in PBMCs from HIV-1-infected patients with or without prior ART treatments by SECH, as determined by TZA assays and humanized mouse-based virus outgrowth assay. Our study suggests a strategy for the eradication of HIV-1 infection by selectively eliminating the infected cells that are capable of producing new viruses.

The SECH approach can delete HIV-1-infected cells while preserving the uninfected healthy cells. Mechanistically, activation of T cells with a latency reversing agent, IDB, led to caspase activation and cell death signaling in HIV-infected cells, whereas sparing the uninfected cells (Fig. 2a, e). Applying IDB has an unintended effect of inducing antiapoptotic molecules and autophagy, which likely inhibit the killing of HIV-1-infected cells (Fig. 2a). ABT-263 and SAR405 are used to mitigate these unintended effects of IDB to promote cell death in HIV-infected cells. Nevertheless, the induction of antiapoptotic molecules and autophagy by IDB may have the benefit of conferring protection of uninfected cells against killing, thereby improving the specificity of the SECH approach in targeting HIV-1-infected cells. An epigenetic modifier, JQ1, could be included in SECH to synergize with IDB for HIV-1 reactivation to promote the clearance of HIV-1-producing cells. Preferential killing of HIV-1-infected cells and protection of uninfected cells are important for the selectivity and safety of this SECH method, resulting in the eradication of HIV reservoir while preserving a normal immune cell repertoire.

Although the agents for SECH can cause rapid and specific clearance of HIV-1-infected cells in vitro (Figs. 2e, 6 and 7), the efficacy in vivo is expected to be lower. Indeed, we observed that clearance of HIV-1 reservoirs in different sets of HIV-1-infected Hu-HSC mice ranged from 40 to 70% (Figs. 3–5 and Supplementary Fig. 5). The pharmacokinetics for these drugs in vivo and the duration of effective drug concentrations present in different tissues remain to be characterized. This will help to determine the optimal doses and frequency of treatments. Inclusion of epigenetic modifier JQ1 further increased HIV-1 reactivation and clearance (Fig. 5, Supplementary Fig. 8a), suggesting that increased virus reactivation can improve the success rate of viral clearance by SECH. Therefore, promoting virus reactivation represents one effective way for improving the success rate of SECH in HIV-1 clearance in vivo. Testing alternative latency reversal drugs may reveal better approaches for virus reactivation to facilitate the clearance of HIV-1 reservoirs.

Autophagy is essential for the protection of long-term maintenance of memory T cells and memory B cells^25–28,58^, and promoting the longevity of other cell types as well^59^. It has been shown that autophagy can regulate HIV-1 replication during persistent infection^60,61^. We found that autophagy did not affect HIV-1 reverse transcription, integration into host genome or reactivation. Rather, inhibition of autophagy promoted caspase activation and cell death induced by virus reactivation with IDB (Fig. 1h, i and Fig. 2b–d). Suppressing autophagy would likely remove a major protective mechanism for memory T cells harboring latent HIV-1, thereby unleashing the cell death machinery triggered by viral replication and by addition of apoptosis inducers.

Despite enormous challenges, the development of HIV-1 vaccines has shown great promises in inducing immune protection against the virus^62,63^. Broad-spectrum neutralization antibodies are valuable in inhibiting viremia by neutralizing HIV-1^64^. Use of CAR T cells to target HIV-1 proteins can suppress HIV-1 infections in humanized mice or patient samples^65,66^. In addition to blocking viral fusion with cell membranes and integration into host genome, broad neutralizing antibodies may be used to replace agents for ART to prevent new infections during SECH treatments.

We show a SECH approach in a humanized mouse model to eradicate the HIV-1 reservoir by a combination of latency reversal, inhibition of autophagy, promotion of apoptosis, and blocking of new rounds of viral replication by ART. Continuous SECH treatments via the oral route can safely and effectively reduce and clear HIV-1 reservoirs established in humanized mice. Moreover, treating PBMCs from HIV-1 patients by SECH led to the successful clearance of HIV-1 infections. Our study suggests a cure strategy for treating HIV-1 infections by selectively eliminating host cells harboring replication-competent HIV-1.

## Methods

### Flow cytometry

The following antibodies from Biolegend were used for flow cytometry: pacific blue-anti-mouse CD45 (1:100, 103126, clone 30-F11,) APC-anti-human CD45 (1:100, 304012, clone HI30), pacific blue-anti-human CD19 (1:100, 302232, clone HIB19), PE-anti-human CD4 (1:100, 317414, clone OKT4), APC/Fire-750-anti-human CD8 (1:100, 34474, clone SK1), FITC-anti-human CD56 (1:100, 392413, clone QA17A16), pacific blue-anti-human CD19 (1:100, 302232, clone HIB19), pacific blue-anti-human CD3 (1:100, 300329, clone HIT3a), PE/Cy7-anti-HLA-HLA-DR, DP, DQ (1:100, 361708, clone Tü39), PerCP/Cy5.5-anti-human CD11b (1:100, 301327, clone ICRF44), APC/Fire-750-anti-human CD163 (1:100, 333633, clone GHI/61), PE-anti-human CD123 (1:100, 306005, clone 6H6), Alexa Fluor 488 anti-human CD11c (1:100, 301618, clone 3.9), PerCP/Cy5.5-anti-human CD3 (1:100, 300328, clone HIT3a), PerCP/Cy5.5-anti-human CD123 (1:100, 306016, clone 6H6), FITC-anti-mouse CD45 (1:100, 103108, clone 30-F11), PE/Cy7-anti-human CD197 (CCR7) (1:100, 353226, clone G043H7), PE-anti-human CD45RO (1:100, 304244, clone UCHL1), FITC-anti-human CD45RA (1:100, 304148, clone HI100) and Alexa Fluor 488 anti-CD68 (1:30, 333812, clone Y1/82 A), PerCP/Cy5.5-anti-human CD19 (1:100, 302230, clone HIB19), PE-anti-human CD27 (1:100, 356406, clone M-T271), and pacific blue-anti-human IgD (1:100, 348224, clone IA6-2). PE-anti-human CD3 (1:100, 556612, clone SP34) and V50-anti-human CD4 (1:100, 560345, clone RPA-T4) were from BD Biosciences. The cells stained with indicated antibodies were analyzed using a BD LSR II flow cytometer (BD Biosciences). To detect HIV-1 p24, spleen cells from Hu-HSC mice were stimulated with 5 μg/ml PHA (Sigma) and 6 ng/ml IL-2 (Biolegend). The cells were stained for T-cell markers, followed by fixation and permeabilization using the Cytofix/Cytoperm buffer (BD Bioscience) and intracellular staining with PE-conjugated anti-p24 (1:30, 6604667, clone KC57, Beckman Coulter). The cells were analyzed by flow cytometry using a BD LSR II flow cytometer (BD Bioscience) and FlowJo software (version 10.5.3, BD Bioscience).

### T-cell isolation and infection with HIV-1

PBMCs of anonymous healthy donors from the Gulf Coast Blood Center were used to purify CD4^+^ T cells with anti-CD4 MACS beads (Miltenyi Biotec). CD3^+^CD4^+^CD45RA^+^CD45RO^−^CCR7^+^ naive T cells, CD3^+^CD4^+^CD45RA^−^CD45RO^+^CCR7^+^ CMT and CD3^+^CD4^+^CD45RA^−^CD45RO^+^CCR7^−^ EMT were sorted using a BD FACSAria flow cytometer (BD Bioscience). Naive CD4^+^ T cells were stimulated with 5 μg/ml PHA and 6 ng/ml IL-2 in Rosewell Park Memorial Institute (RPMI) complete medium for 2 days to generate activated CD4^+^ T cells. Sorted T-cell subsets or activated T cells were infected with HIV-1 CXCR4-tropic NL4-3 or CCR5-tropic AD8 (both virus clones were obtained from the NIH AIDS Reagent Program) at the indicated MOI for 2 h. The cells were washed with PBS and cultured in RPMI complete medium containing 30 nm CCL19 (Biolegend) and 0.3 ng/ml IL-2 (Biolegend) and for 4 days to establish latent HIV infection as described^29^.

For gene silencing, CMT were transfected with 100 nm siRNA targeting Atg7 or control siRNA (Dharmacon) using the Neon Transfection System at 2150 volts with one pulse of 20 ms (Life Technologies). Virtually all cells were transfected using this condition with a fluorescently labeled siRNA. Gene silencing was confirmed by Western blot (Fig. 1a). The cells were infected with 0.1 MOI of HIV-1 (HIV-1 NL4-3) at 37 °C for 2 h. The cells were then cultured with CCL19 as above to establish HIV latent infection.

Naive T cells, activated T cells, CMT or EMT with or without infection with HIV-1 (NL4-3, 1 MOI) were cultured for 4 days in the presence of 30 nm CCL19 and 0.3 ng/ml IL-2 for 4 days to establish latent HIV infection. The cells with or without HIV-1 infections were stimulated with 0.1 μm IDB (ENZO Life Sciences) in the presence of 0.2 μm ABT-263 (Adooq Bioscience), 2 μm SAR405 (MedChemExpress), or 10 μm CQ (Sigma) as indicated for 48 h. The cells were then incubated with 1 μm FITC-DEVD-FMK (Biovision), followed by staining with APC-Annexin V (Biolegend) and intracellular staining with PE-anti-HIV p24. The cells were analyzed by flow cytometry. The percentage of cell death was calculated by the loss of live cells negative for annexin V and DEVD staining by comparing treated and untreated groups: (untreated−treated)/untreated × 100%.

To determine the specificity in the killing of HIV-1 infected cells, uninfected CD4 cells were labeled with CellTrace Violet dye (ThermoFisher Scientific) and mixed with HIV-1-infected cells at the ratio of 1:1. The cells were resuspended in RIPMI complete medium containing 0.2 μm BMS-626529, with 0.1 μm IDB, 0.2 μm ABT-263, and 2 μm SAR405 as indicated for 48 h. The cells were incubated with 1 μm FITC-DEVD-FMK, followed by staining with APC-Annexin and analyses by flow cytometry.

CD4 cells with or without HIV-1 infection cultured with or without 1 μm SAR405 for 12 hours and fixed in the 4% formaldehyde (ThermoFisher Scientific). The fixed cells were then incubated with rabbit anti-LC3A/B antibody (1:100, 12741 s, Cell Signaling Technology), followed by staining with Alexa Fluor 594 donkey anti-rabbit IgG (1:5000, A21207, Thermo Fisher Scientific). LC3 punctate in the cells were quantified under a fluorescent microscope.

### Detection of HIV-1 DNA products of reverse transcription and proviral DNA integration

CMT at different hours after HIV-1 infection were used for PCR to detect HIV-1 reverse transcription products as described^36^, including early *R/U5* product, sense primer (5′-GGCTAACTAGGGAACCCACTG-3′), antisense primer (5′-CTGCTAGAGATTTTCCACACTGAC-3′), and late *LTR-gag* product, sense primer (5′-CAGATATCCACTGACCTTTGG-3′), antisense primer (5′-GCTTAATACTGACGCTCTCGCA-3′). β-globin was detected by PCR with β-globin forward, 5′-CCCTTGGACCCAGAGGTTCT-3′ and β-globin reverse, 5′-CGAGCACTTTCTTGCCATGA-3′. *R/U5* and *LTR-gag* PCRs were normalized against β-globin. Genomic DNA from CD4^+^ T cells at 4 days after initial HIV-1 infections was used for real-time PCR for *Alu-gag* using the following primers^37^: *Alu* forward primer (5′-GCCTCCAAAGTGCTGGGATTACAG-3′) and *gag* reverse primer (5′-GTTCCTGCTATGTCACTTCC-3′). The relative levels of *Alu*-*gag* were normalized against β-globin.

### Western blot

PBMC CD4^+^ T cells with or without infection by HIV-1 cultured for 4 days with CCL19 as in Fig. 1a. The cells were then cultured in the absence or presence of 0.1 μm IDB for 24 h. The cells were lysed in lysis buffer containing 50 mm HEPES, pH 7.5, 150 mm NaCl, 1 mm ethylenediaminetetraacetic acid (EDTA), 1% Triton X-100, 1X protease inhibitor mixture (Roche Applied Science) and 10 μm*Benzyloxycarbonyl*-Val-Ala-Asp (OMe) fluoromethylketone (zVAD-FMK, ENZO Life Sciences). The cell lysates were determined by the Bradford assay (Bio-Rad). Samples were used for sodium dodecyl sulfate polyacrylamide gel electrophoresis and western blot analyses by probing with different antibodies: monoclonal antibodies to caspase-9 (1:1000, M054-3, clone 5B4) and caspase-7 (1:1000, M053-3, clone 4G2) from Medical & Biological Laboratories; polyclonal rabbit antibodies to Atg7 (1:1000, 2631 S), cleaved caspase-9 (1:1000, 52873 s), caspase-3 (1:1000, 9665 s); cleaved caspase-3 (1:1000, 9501 s), caspase-6 (1:1000, 9762 s), Mcl-1 (1:1000, 5453 s), Bcl-2 (1:1000, 4223 s), Bcl-xL (1:1000, 2762 s), Bak (1:1000, 12105 s), Bax (1:1000, 2772 s), LC3 (1:1000, 4108 s) from Cell Signaling Technology and monoclonal antibody to β-Actin (1:50,000, sc-47778, clone C4) from Santa Cruz Biotechnology. The blots were them incubated with HRP-conjugated goat anti-mouse IgG1 (1:50,000, 1070-05, Southern Biotech) or HRP-conjugated goat anti-Rabbit IgG (1:50,000, ab6721, Abcam) and developed using SuperSignal West Dura Extended Duration Substrate (ThermoFisher).

### Quantification of HIV-1 mRNA and DNA

HIV-1 mRNA was measured by RT-PCR similar to the described protocol^67^. *LTR-gag* was amplified with forward primer (LTR-GAG-AF), 5′-GATCTCTCGACGCAGGACTC-3′ and reverse primer (LTR-GAG-AR), 5′-CGCTTAATACCGACGCTCTC-3′, and detected with the *LTR-gag* probe, 5HEX/CCAGTCGCC/ZEN/GCCCCTCGCCTC/3IABkFQ. HIV-1 *pol-1* was amplified with POL-1 forward primer (POL-1-AF), 5′-AGCAGGAAGATGGCCAGTAA-3′ and reverse primer (POL-1-AR), 5′-GGATTGTAGGGAATGCCAAA-3′, and detected with the *pol-1* probe FAM/CCCACCAAC/ZEN/ARGCRGCCTTAACYG/3IABKFQ in iTaq Universal Probes Supermix (Bio-Rad). The reaction was carried out by iTaq Universal Probes Supermix (Bio-Rad) with a QuantStudio 5 Real-Time PCR System and analyzed with the QuantStudio Design and Analysis Software (Applied Biosystem). Cycle threshold (Ct) values were calibrated using standard curve generated with the standard samples with known amounts of viral copies.

Spleen cells were isolated and red blood cells were lysed with ammonium chloride lysing buffer (0.15 m NH4Cl, 10 mm KHCO3, 0.1 mm EDTA). Bone marrow cells were collected from femur and tibia. The cells were used for mRNA preparation with MagMAX-96 for Microarrays Total RNA Isolation Kit (ThermoFisher Scientific). Tissues (50 mg) were homogenized with Precellys Lysing Kit (Cayman Chemical). mRNA was extracted and converted to cDNA using the SuperScript VILO cDNA Synthesis Kit (Thermo Fisher Scientific), followed by real-time PCR for Pol-1 and LTR-gag as above. Quantity of viral DNA was performed by real-time PCR of genomic DNA from the cells with the described primers as described^68^: forward primer, 5′-GGTCTCTCTGGTTAGACCAGAT-3′ and reverse primer, AGATTTTCCACACTG and probe 5′-6FAMAGTAGTGTGTGCCCGTCTGTT-TAMRA-3′) for amplification of an HIV-1 *LTR* sequence.

### Generation of humanized mice for HIV-1 infection and cure studies

NSG-SGM3 mice (stock no: 013062, The Jackson Laboratory) were maintained on a 12-hour light/dark cycle with the temperature (22 °C) and humanity (40–60%) controlled environment in the specific-pathogen-free barrier animal facility at the Houston Methodist Research Institute. Newborn male and female mice were injected intrahepatically with CD34^+^ human stem cells (5 × 10^4^/mouse; AllCells LLC). Three months later, reconstitution of human immune cells in mouse peripheral blood was determined by flow cytometry. Both male and female mice were used for the experiments. HIV-1-infection and cure experiments were performed in Biosafety Level 2 facilities in the Houston Methodist Research Institute. These human CD34^+^ cell-reconstituted mice (Hu-HSC mice) were then injected intraperitoneally with HIV-1 AD8 (1000 pfu/mouse). Seven days later, peripheral blood was collected and RNA was extracted using the MagMAX-96 Blood RNA Isolation Kit (Thermo Fisher Scientific). RNA was converted to cDNA using the SuperScript VILO cDNA Synthesis Kit (Thermo Fisher Scientific), followed by real-time PCR for *LTR-gag* and *Pol-1* as above. Experiments were performed according to federal and institutional guidelines and with the approval of the Institutional Animal Care and Use Committee of the Houston Methodist Research Institute.

A total of eight sets of experiments were performed to establish HIV-1 infections and treatments using HSC-Hu mice, including three sets of experiments to test the reconstitution of NSG-SGM3 mice with human CD34^+^ stem cells and the establishment of HIV-1 infections in these mice, two sets of experiments to determine the dose of drugs that could be safely used in C57BL/6 mice and NSG-SGM3-derived HSC-Hu mice, one set of experiments in Supplementary Fig. 5c–g to test the SECH procedure after initial ART treatments, one set of experiments with SECH treatments in Figs. 3–4 and Supplementary Figs. 4–7, and one set of SECH treatments with or without JQ1 in Fig. 5 and Supplementary Fig. 8. For SECH treatments, raltegravir (20 mg/kg b.w.), BMS-663068 (20 mg/kg b.w., Adooq Bioscience), IDB (2.5 mg/kg b.w.), ABT-263 (50 mg/kg b.w.), and SAR405 (50 mg/kg b.w.) with or without JQ1 (25 mg/kg b.w.) were formulated in the solvent containing 10% ethanol, 30% polyethylene glycol 400 (Sigma), and 60% Phosal 50 PG (Fisher Scientific), and administered by oral gavage once every 2 days. Raltegravir and BMS-663068 (20 mg/kg b.w.) alone were also administered on the alternate days. For the ART control group, raltegravir and BMS-663068 (20 mg/kg b.w.) were given daily. In addition, tablets containing non-steroid anti-inflammatory carprofen^69^ (2 mg in each 5 g tablet, Bio-Serv) were supplied together with regular diet pellets to mice. During treatments, mice were monitored daily for body weight, food consumption, and mobilities. At the end of experiment, histological analysis by hematoxylin and eosin staining was carried out in the major vital organs (brain, liver, lung, and kidney).

Peripheral blood was collected at different intervals to detect HIV-1 mRNA by RT-PCR. After treatments, spleen and bone marrow were collected for analyses by RT-PCR, virus outgrowth assay and intracellular staining for HIV-1 p24. Some mice were kept for an additional 2 months with no treatments. Virus clearance was determined in the spleen, bone marrow by RT-PCR, TZA assay, and p24 intracellular staining. RT-PCR was also performed to detect HIV-1 mRNA in the lung, liver, and kidney for some mice.

### TZA assay

TZM-bl cells^70^ obtained from the NIH AIDS Reagent Program were cultured in 96-well plates (60,000 cells/well) for 24 hours. TZA assay was performed similar to described procedures^50^. Spleen and bone marrow cells (5 × 10^6^/sample) from Hu-HSC mice were stimulated with anti-CD3- and anti-D28-Dynalbeads for 48 h, followed by co-cultured with TZM-bl cells for another 48 h in the presence of 5 μg/ml PHA, 0.1 μg/ml LPS and 100 nm CpG. Beta-galactosidase activity was determined using the Beta-Glo Assay System (Promega). This virus outgrowth assay can detect between 1 and 400 pfu of HIV-1 in a linear fashion, and the virus titers in the samples were calculated based on HIV-1 standard titration.

### Hu-HSC mouse-based virus outgrowth assay in vivo (hmVOA)

Spleen or bone marrow cells (5 × 10^6^/sample) from HIV-1-infected Hu-HSC mice treated by SECH or ART were transferred into uninfected recipient Hu-HSC mice intravenously similar to previously described procedures^54^. HIV-1 in the peripheral blood of recipient mice was determined 4 weeks later by RT-PCR. PBMCs from HIV-1-infected patients (3 × 10^6^/sample) with or without SECH treatments in vitro were also transferred into Hu-HSC mice intravenously. HIV-1 in the mouse peripheral blood was determined by RT-PCR.

### Treatment of PBMCs from HIV-1-infected patients

PBMCs from HIV-1 patients were resuspended in RPMI complete medium containing and 0.3 ng/ml IL-2 and 50 ng/ml M-CSF and cultured with 0.2 μm BMS-626529, 0.2 μm raltegravir, 25 nm IDB, 20 nm ABT-263, 0.1 μm SAR405, and 0.25 μm JQ1 for 2 days as one cycle of treatments. The cells were then washed and cultures in the same medium for next cycle of culture. After seven cycles of culture, RNA was prepared from the cells for RT-PCR analyses of HIV-1. For hmVOA, the cells were incubated with biotin-conjugated anti-CD8 and BioMag streptavidin beads (Sigma) to deplete CD8^+^ T cells. The cells (3 × 10^6^) were then adoptively transfer into uninfected Hu-HSC mice intravenously for hmVOA as above. Experiments with ART-naive patients were performed according to federal and institutional guidelines, with written informed consents and the approval of the Institutional Review Boards of University of Texas Health Science Center at Houston and Houston Methodist Research Institute. Experiments with de-identified samples from ART-treated patients were performed according to federal and institutional guidelines with the approval of the Institutional Review Board of the Houston Methodist Research Institute.

### Statistical analyses

GraphPad Prism 8 was used for statistical analyses. Data were presented as the mean ± SD and *p* values were determined by two-tailed Student’s *t* test. Mann–Whitney test was also used for analyzing humanized mice and patient PBMCs. Significant statistical differences (*p < 0.05 or **p < 0.01) are indicated.

### Reporting summary

Further information on research design is available in the Nature Research Reporting Summary linked to this article.

## Supplementary information

Supplementary Information

Reporting Summary

## Data Availability

Source data are provided with this paper. All the other data supporting the findings of this study are available within the article and are available from the corresponding author.

## Source data

Source Data
