# Author Correction: Clearance of HIV infection by selective elimination of host cells capable of producing HIV

**DOI:** 10.1038/s41467-020-20218-9

**Published:** 2020-12-02

**Authors:** Min Li, Wei Liu, Tonya Bauch, Edward A. Graviss, Roberto C. Arduino, Jason T. Kimata, Min Chen, Jin Wang

**Affiliations:** 1 Immunobiology and Transplant Science Center, Houston Methodist Research Institute, Houston, TX 77030 USA; 2 Department of Pathology and Genomic Medicine, Houston Methodist Research Institute, Houston, TX 77030 USA; 3 Division of Infectious Diseases, Department of Internal Medicine, McGovern Medical School at The University of Texas Health Science Center, Houston, TX 77030 USA; 4 Department of Molecular Virology and Microbiology, Baylor College of Medicine, Houston, TX 77030 USA; 5 Department of Pathology and Immunology, Baylor College of Medicine, Houston, TX 77030 USA; 6 Department of Surgery, Weill Cornell Medical College, Cornell University, New York, NY 10065 USA

Correction to: *Nature Communications* 10.1038/s41467-020-17753-w, published online 13 August 2020.

The original version of this Article contained errors in the labelling of the *y*-axis scales in Figs. 3e–g and 4a. In Fig. 3e, the *y*-axis scale was incorrectly labelled from ‘10^2^’ to ‘10^9^’, rather than the correct ‘10^0^’ to ‘10^6^’. In Fig. 3f, the *y*-axis scale was incorrectly labelled from ‘10^2^’ to ‘10^7^’ in the left panel and from ‘10^2^’ to ‘10^8^’ in the right panel, rather than the correct ‘10^0^’ to ‘10^4^’. In Fig. 3g, the *y*-axis scale was incorrectly labelled from ‘10^2^’ to ‘10^5^’ in both panels, rather than the correct ‘10^0^’ to ‘10^3^’. In Fig. 4a, the *y*-axis scale was incorrectly labelled from ‘10^0^’ to ‘10^9^’ in the right panel, rather than the correct ‘10^0^’ to ‘10^6^’. These errors have been corrected in both the PDF and HTML versions of the Article.

