# Supplementary material for: Clearance of HIV infection by selective elimination of host cells capable of producing HIV
Source: Nat Commun. 2020 Aug 13;11:4051. doi: 10.1038/s41467-020-17753-w (PMC7426846; doi:10.1038/s41467-020-17753-w)
Supplement: Supplementary file 1 — Supplementary Information [file 41467_2020_17753_MOESM1_ESM.pdf]

Supplementary Information for:

Clearance of HIV Infection by Selective Elimination of Host Cells Capable of Producing HIV

Min Li[1], Wei Liu[1], Tonya Bauch[1], Edward A. Graviss[2], Roberto C. Arduino[3], Jason T. Kimata[4], Min Chen[5], and Jin Wang[1,6*]

[1]Immunobiology and Transplant Science Center, Houston Methodist Research Institute, Houston, TX 77030, USA

[2]Department of Pathology and Genomic Medicine, Houston Methodist Research Institute, Houston, TX 77030, USA

[3]Division of Infectious Diseases, Department of Internal Medicine, McGovern Medical School at The University of Texas Health Science Center, Houston, TX 77030, USA

[4]Department of Molecular Virology and Microbiology, Baylor College of Medicine, Houston, TX 77030, USA

[5]Department of Pathology and Immunology, Baylor College of Medicine, Houston, TX 77030, USA

[6]Department of Surgery, Weill Cornell Medical College, Cornell University, New York, NY 10065, USA

*Correspondence: jinwang@houstonmethodist.org

This document includes Supplementary Figures 1-9, and Supplementary Tables 1-3.

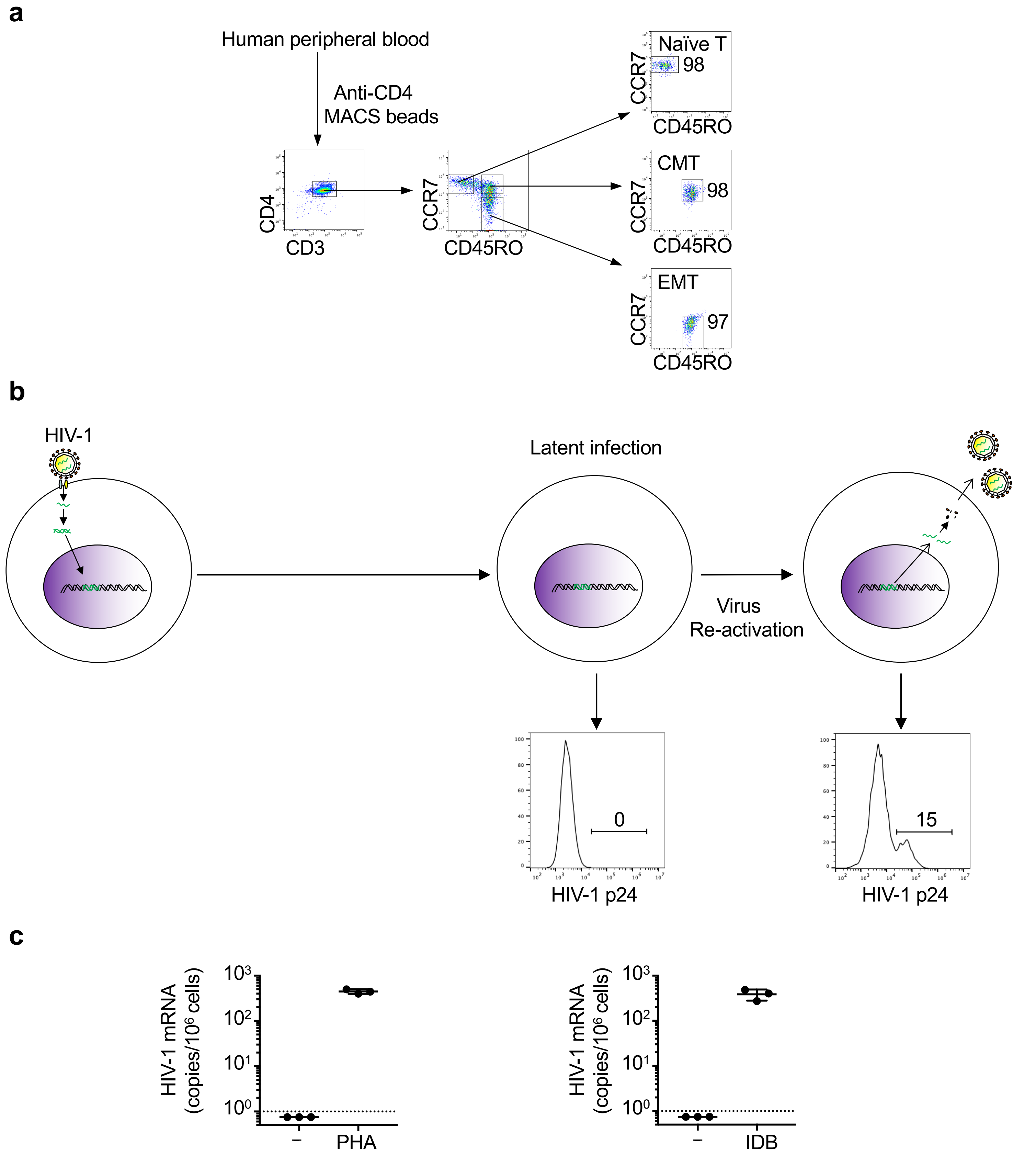

Supplementary Figure 1. Establishment of HIV-1 latent infection in sorted T cell.
(**a**) $CD4^+$ T cells were purified from human PBMCs using anti-CD4-MACS beads (Miltenyi Biotec). $CD3^+CD4^+CD45RA^+CD45RO^-CCR7^+$ naïve T cells, $CD3^+CD4^+CD45RA^-CD45RO^+CCR7^+$ central memory T cells (CMT) and $CD3^+CD4^+CD45RA^-CD45RO^+CCR7^-$ effector memory T cells (EMT) were sorted by flow cytometry.
(**b**) CMT were infected with HIV-1 and cultured for 4 days with CCL19 to establish latent infections. Latently infected cells were stimulated with PHA for virus reactivation, followed by intracellular staining for HIV-1 p24.
(c) Latently infected cells were stimulated with PHA or IDB for virus reactivation, followed by RT-PCR analyses. Data are presented as mean ± SD ($n = 3$ biologically independent samples). The dash line indicates detection limit. Source data are provided as a Source Data file.

a

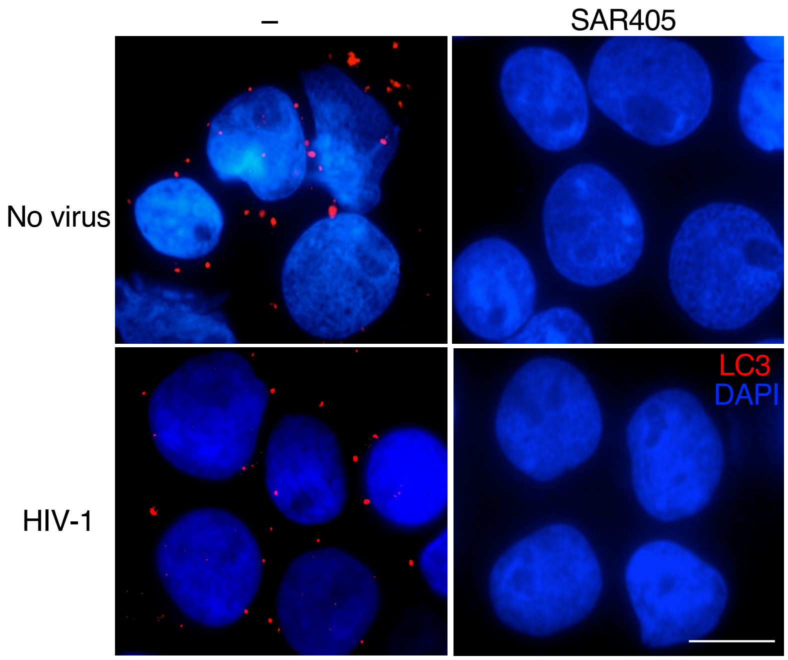

b

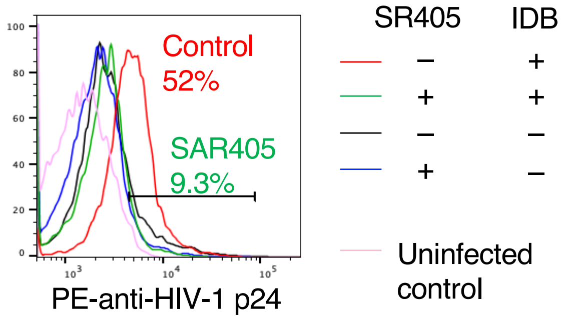

Supplementary Figure 2. Inhibition of autophagy in HIV-1-infected T cells.
(**a**) CMT with or without HIV-1 infection as in Fig. 1 were cultured with or without SAR405 for 12 h, following by immunocytochemistry staining for LC3. The images are representative of two independent experiments. Scale bar, 10 µm.
(**b**) CMT were infected with HIV-1 AD8 (1 MOI). The cells were cultured with CCL19 in the presence or absence of SAR405 for 4 days. The cells were then cultured with or without IDB for 24 h, followed by intracellular staining for HIV-1 p24 and flow cytometry. Percentages of HIV-1 $p24^{+}$ cells are shown.

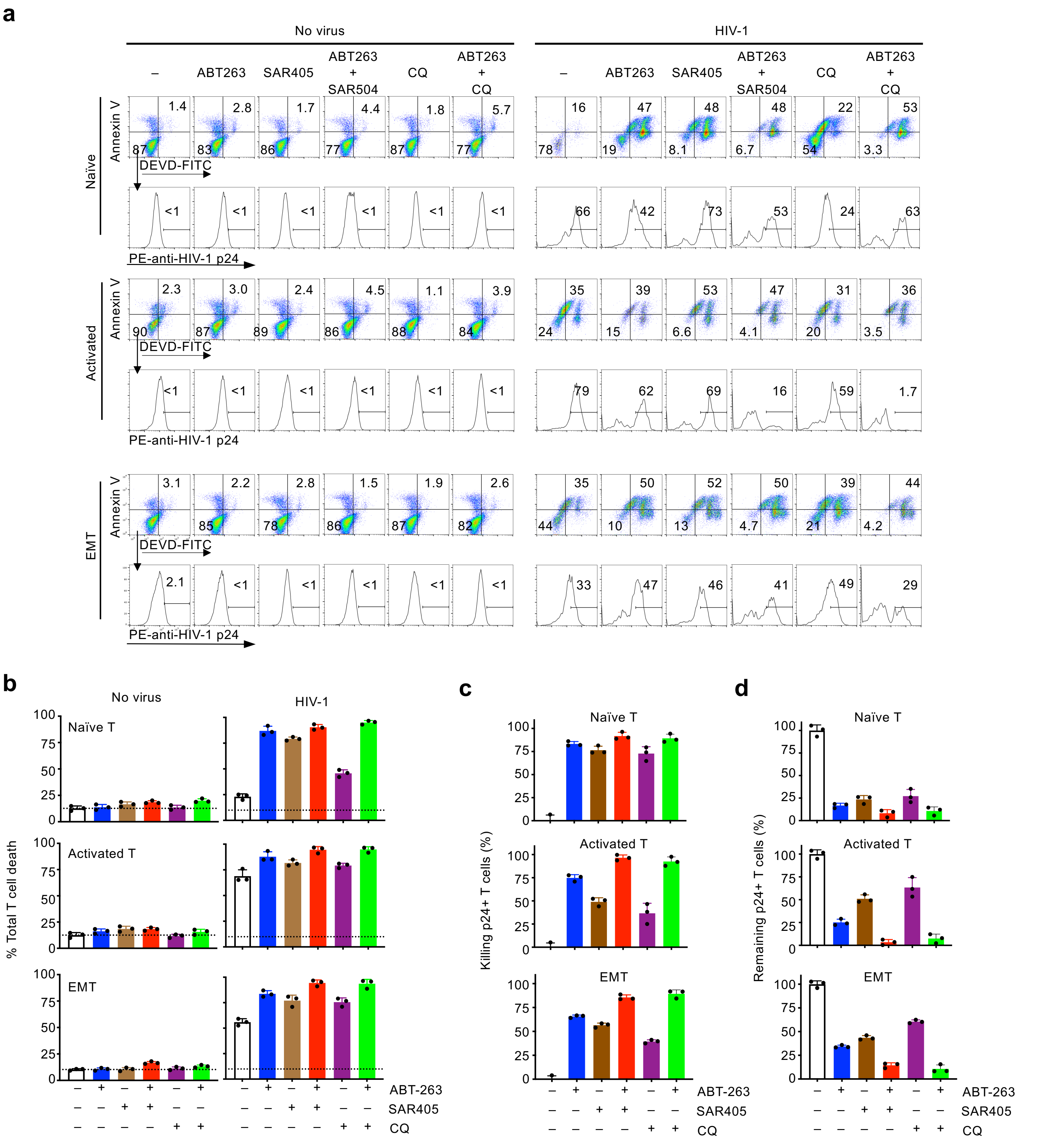

Supplementary Figure 3. Induction of cell death in T cells with or without HIV-1 infections.
(**a**) Naïve T cells, activated T cells, or EMT with or without infection by HIV-1 (NL4-3, 1 MOI) were cultured for 4 days to establish latency as in Fig. 1. The cells were stimulated with 0.1 μM IDB. ABT-263 (0.2 μM), SAR405 (2 μM) and chloroquine (CQ, 10 μM) were added in different samples as indicated. The cells were cultured for 48 h. The cells were then incubated with DEVD-FITC, followed by staining with APC-Annexin V and intracellular staining with PE-anti-HIV p24. The cells were analyzed by flow cytometry.
(**b**) Percentage of cell death among all T cells.
(**c**) Percentage of killing of HIV-1 p24⁺ T cells by indicated combination of agents after IDB-induced virus reactivation.
(**d**) Percentage of remaining HIV-1 p24⁺ viable T cells negative for DEVD-FITC and Annexin V staining after treatments by indicated combinations of ABT-263 and SAR405 or CQ compared to untreated controls.
In b, c and d, data are representative of three biologically independent samples and presented as mean ± SD.
Source data are provided as a Source Data file.

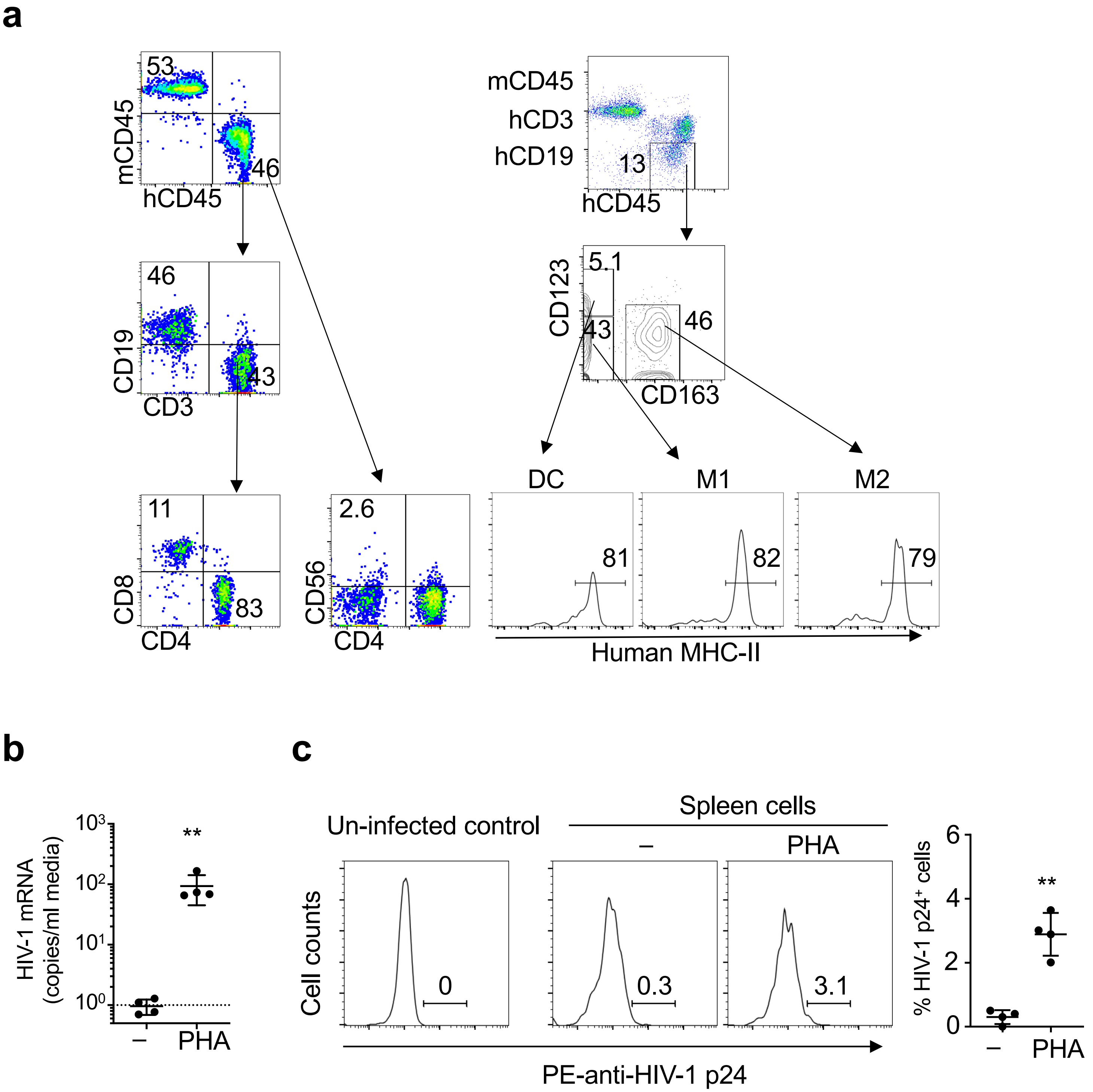

Supplementary Figure 4. Analyses of Hu-HSC mice by flow cytometry.

(a) Flow cytometry analyses of immune cells in the peripheral blood of Hu-HSC mice. NK, nature killer cells; DC, dendritic cells; M1, M1 macrophages; M2, M2 macrophages.

(b) Spleen cells were isolated from Hu-HSC mice ($n = 4$) 10 days post HIV-1 infection and cultured with or without PHA for 24 h. Culture media were collected for HIV-1 mRNA determination by RT-PCR. Data are presented as mean ± SD. PHA *vs.* control, $^{**}p=0.0088$ (unpaired two-tailed *t* test).

(c) Spleen cells treated in (b) were stained for CD4, followed by intracellular staining of HIV-1 p24 and flow cytometry analysis. Increases in $p24^{+}$ cells after PHA stimulation suggest that HIV-1 infections in some cells have established latency. Data are presented as mean ± SD. PHA *vs.* control, $^{**}p = 0.0003$ (unpaired two-tailed *t* test).

Source data are provided as a Source Data file.

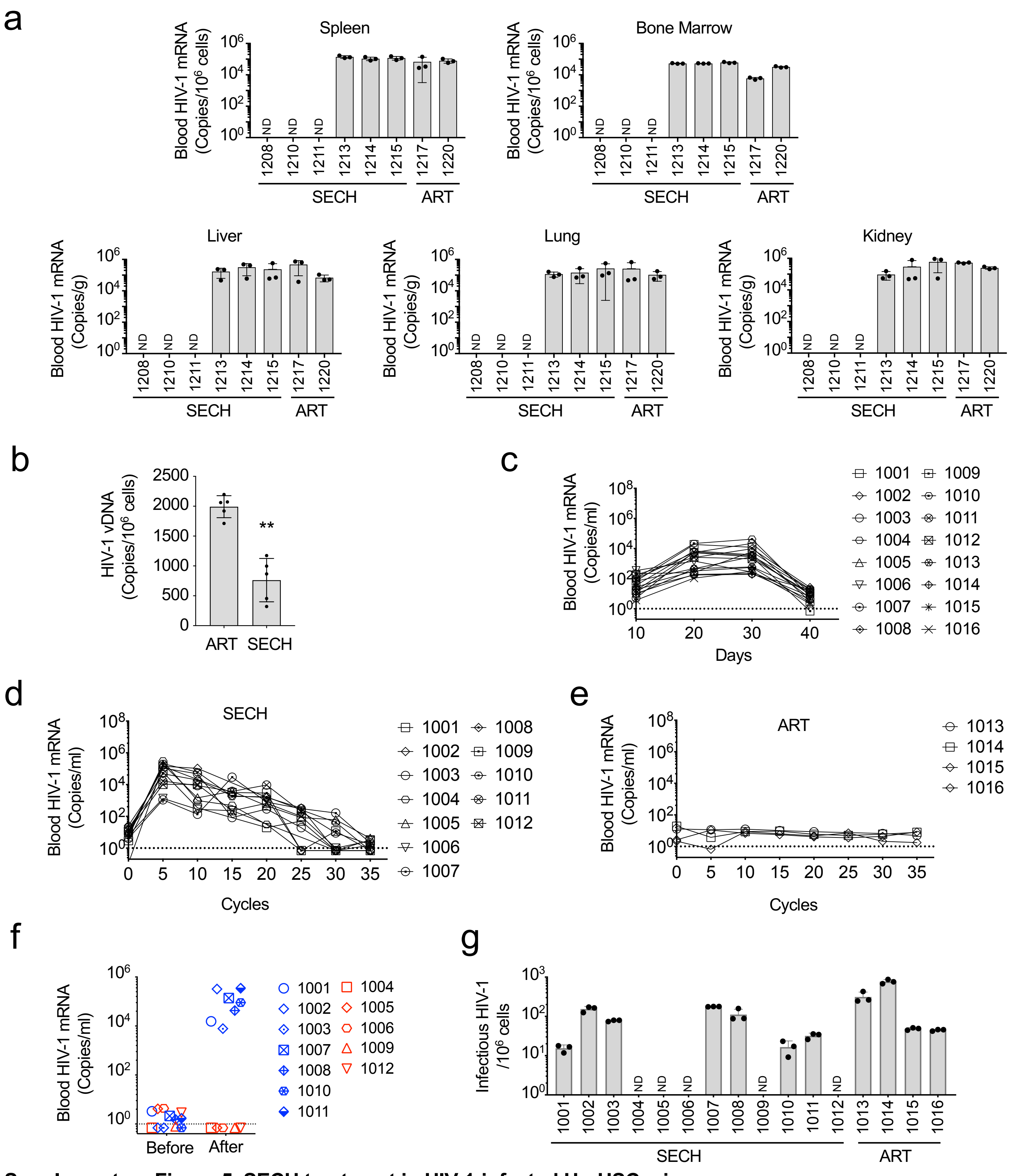

Supplementary Figure 5. **SECH treatment in HIV-1-infected Hu-HSC mice.**
(a) Detection of HIV-1 mRNA in tissues and organs from mice after treatments by SECH or ART.
(b) Determination of HIV-1 genomic DNA in spleen from mice after treatments by SECH ($n = 5$) or ART ($n = 5$). Data are presented as mean ± SD. SECH *vs.* ART: $^{**}p = 0.0001$ (unpaired two-tailed t test).
(c) HIV-1 mRNA in the total peripheral blood was determined by RT-PCR prior to SECH treatment. The dash line indicates detection limit.
(d,e) HIV-1 mRNA in the total peripheral blood was determined by RT-PCR after SECH (d) or ART (e) treatment.
(f) HIV-1 mRNA in the peripheral blood of HIV-1-infected Hu-HSC mice after withdrawal of treatments for 4 weeks.
(g) Infectious HIV-1 in the spleen of mice in (f) was determined by TZA assays. ND, not detectable.
In a and g, biological samples were measured in three technical replicates and data are presented as mean ± SD.
Source data are provided as a Source Data file.

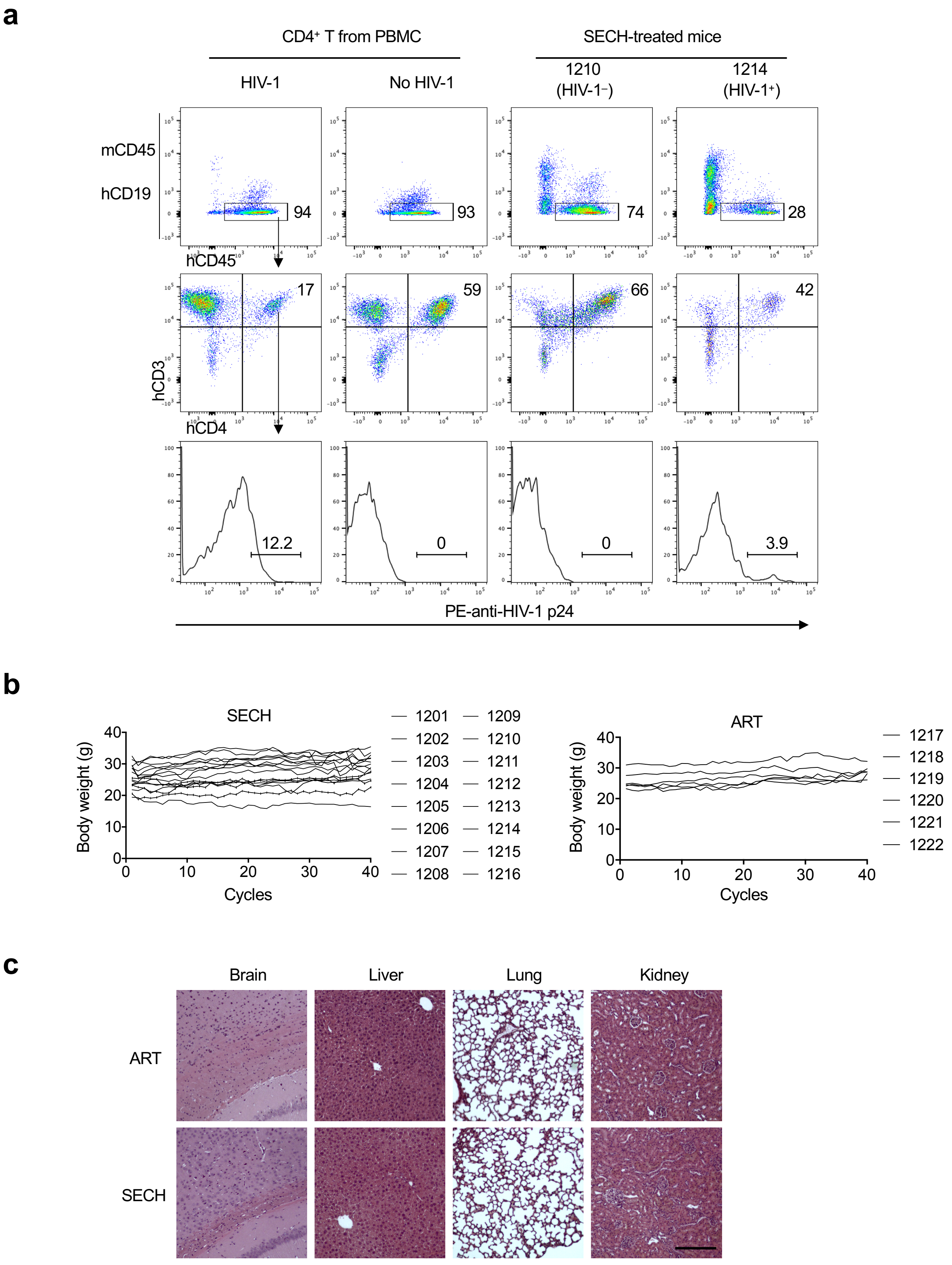

Supplementary Figure 6. Analyses of Hu-HSC mice treat by SECH or ART.

(a) Detection of HIV-1 p24 in T cells from Hu-HSC mice after SECH treatments. Spleen cells of SECH-treated mice were stimulated with PHA plus LPS and CpG. $mCD45^{-}hCD19^{-}hCD45^{+}CD3^{+}CD4^{+}$ human CD4 T cells were gated to determine p24 staining. Human PBMC $CD4^{+}$ T cells with or without HIV-1 infection were used as controls.

(b) Mouse body weight during SECH treatment in Figures 3 and 4.

(c) H&E staining of tissue sections from HIV-infected Hu-HSC mice treated by ART or SECH (representative of 4 independent experiments). Scale bar: 200 µm.

Source data are provided as a Source Data file.

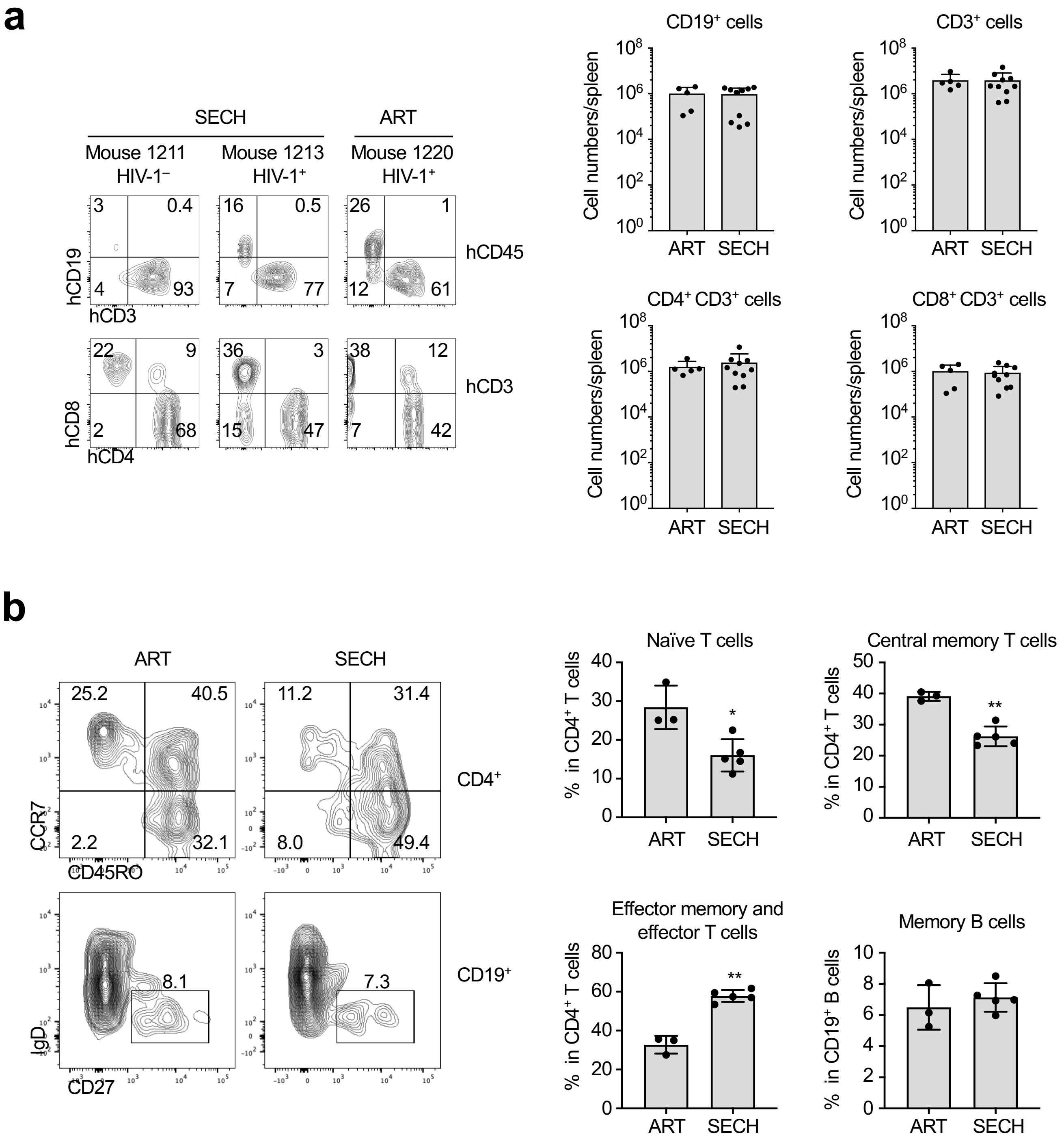

Supplementary Figure 7. Analyses of Hu-HSC mice treated by SECH or ART.

(a) HIV-1-infected Hu-HSC mice were treated by SECH or ART for 40 cycles. After withdrawal of treatments, the types of immune cells from the spleen were determined by flow cytometry (left panel). The number of immune cells in the spleen were shown in right panel (ART, $n = 5$; SECH, $n = 10$). Data are presented as mean ± SD.

(b) HIV-1-infected Hu-HSC mice were treated by SECH or ART for 40 cycles. After withdrawal of treatments, immune memory cells from the spleen were determined flow cytometry (left panel). $CCR7^{+}CCD45RO^{-}$ naïve T cells, $CCR7^{+}CCD45RO^{+}$ central memory T cells, $CCR7^{-}CCD45RO^{+}$ effector memory T cells and $CCR7^{-}CCD45RO^{-}$ effector T cells and $CD19^{+}CD27^{+}$ memory B cells were quantified (right panel). Data are presented as mean ± SD. SECH ($n = 5$) *vs.* ART ($n = 3$): p = 0.0111 (Naïve T), 0.0006 (Central memory T cells), 0.0111 (Effector and effector memory T) and 0.4627 (Memory B cells) as determined by unpaired two-tailed t test.

Source data are provided as a Source Data file.

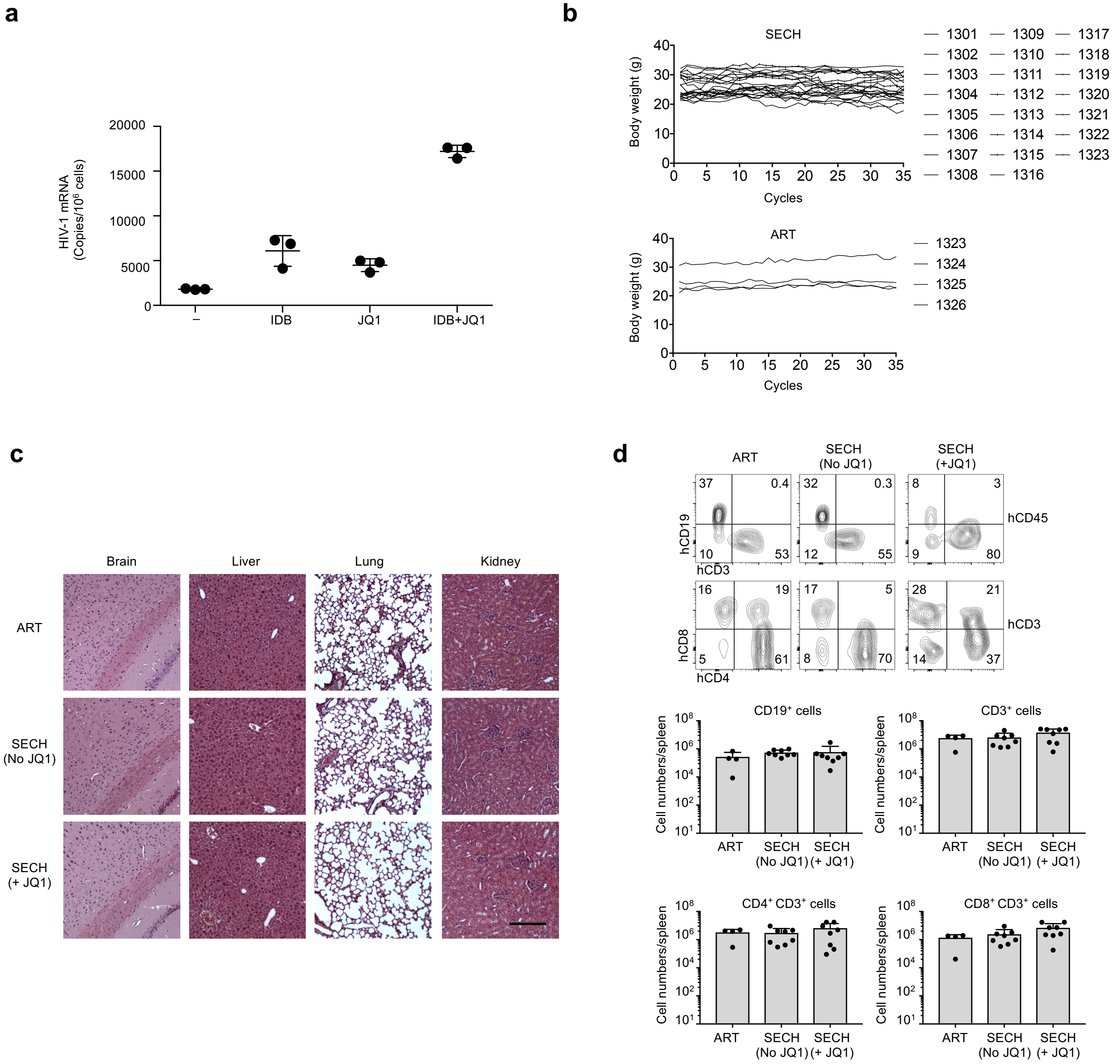

Supplementary Figure 8. SECH treatment in HIV-1-infected Hu-HSC mice.
(**a**) Latently infected CD4 T cells were cultured with IDB, JQ1 or both for 24 h. HIV-1 mRNA was measured by RT-PCR. Data are presented as mean ± SD (*n* = 3 biologically independent samples).
(**b**) Mouse body weight during SECH treatments in Figure 5.
(**c**) H&E staining of tissue sections from HIV-infected Hu-HSC mice treated by ART or SECH. Data are representative of 4 independent experiments. Scale bar: 200 μm.
(**d**) Flow cytometry analyses of immune cells in the spleen of Hu-HSC mice treated by ART (*n* = 4), SECH (*n* = 8) or SECH+JQ1 (*n* = 8). Data are presented as mean ± SD.
Source data are provided as a Source Data file.

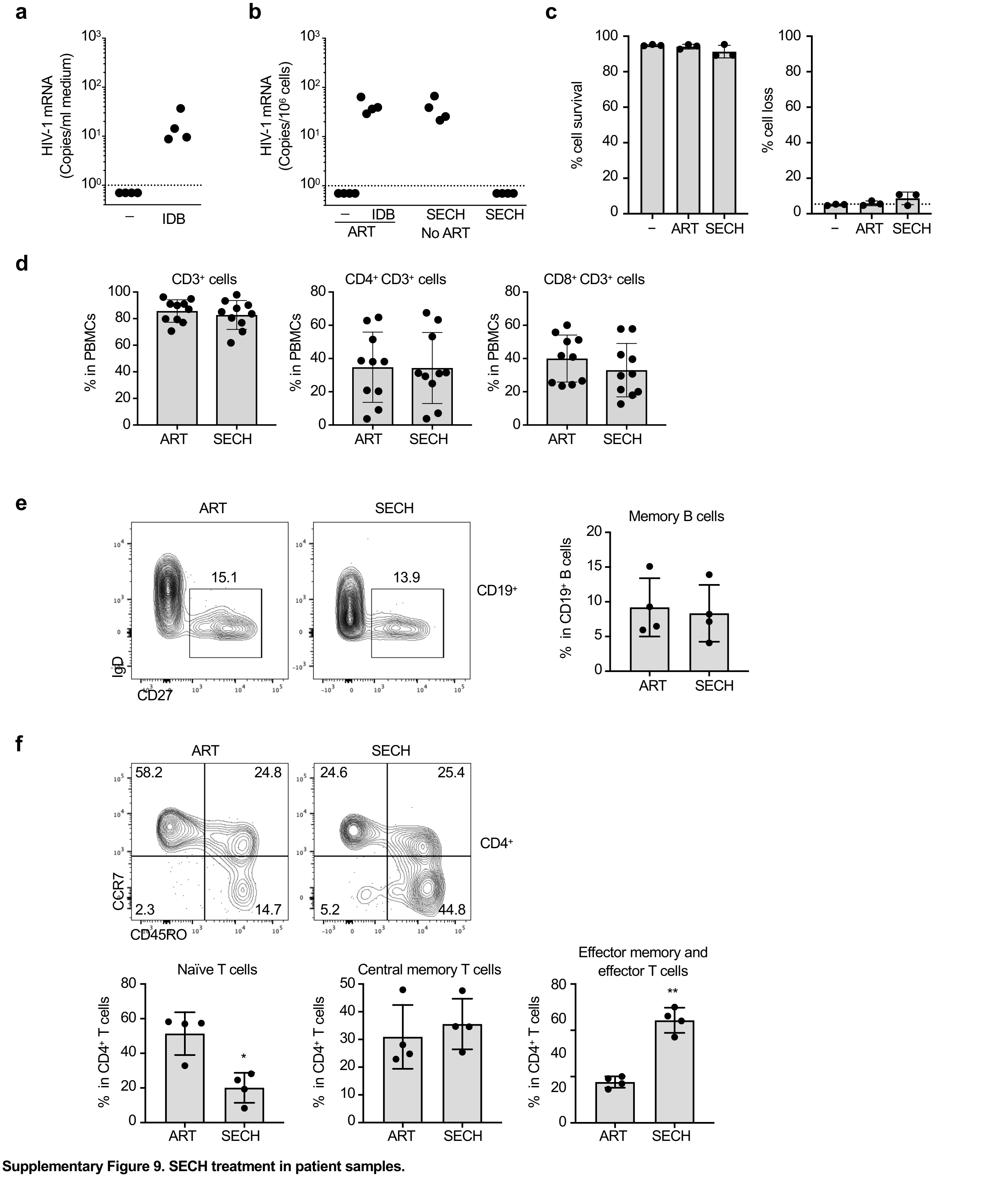

Supplementary Figure 9. SECH treatment in patient samples.

(**a**) Determination of HIV-1 mRNA in culture media from PBMCs of ART-treated patients ($n = 4$) by pol RT-PCR.

(**b**) PBMCs of ART-treated patients were treated by ART, SECH, SECH without antivirus drugs, respectively, for 7 cycles, cell associated HIV-1 mRNA was determined by pol RT-PCR.

(c) The percentage of cell survival and loss of live cells after one cycle of treatments by SECH or ART($n = 3$ biologically independent samples). Data are presented as mean ± SD**.**

(**d**) The percentage of immune cells in PBMCs from ART-treated patients after treatment with ART or SECH ($n = 10$ patients). Data are presented as mean ± SD.

(**e**) $CD19^{+}IgD^{-}CD27^{+}$ memory B cells in PBMCs from HIV-1⁺ patients ($n = 4$) after 1 cycle of treatment by ART or SECH. Data are presented as mean ± SD.

(**f**) PBMCs purified from HIV-1⁺ patients ($n = 4$) were treated by SECH or ART for 7 cycles. T cells were determined by flow cytometry. Data are presented as mean ± SD. SECH *vs.* ART: p = 0.0060 (Naïve T cells), 0.5502 (Central memory T cells) and 0.0001 (Effector memory and effector T cells) as determined by unpaired two-tailed *t* test.

Source data are provided as a Source Data file.

Supplementary Table 1. HIV-1-infected Hu-HSC mice used for SECH treatments.

Mouse ID	SEX	Treatment	% mCD45	% hCD45	hCD45+			hCD45+		hCD45+CD3-CD19-			HIV-1 detection			
					%B cell	%CD3	%NK	%CD4	%CD8	%DC	%M1	%M2	Withdrawl (RT-PCR)	TZA	hmVOA	HIV clearance
1201	Male	SECH	61.1	36.3	79.9	14.2	1.0	9.3	3.8	1.3	47.0	32.9	HIV–	HIV–	HIV–	HIV–
1202	Male	SECH	51.6	44.8	78.0	12.2	1.6	9.2	2.3	1.8	52.6	30.2	HIV–	HIV–	HIV–	HIV–
1203	Male	SECH	75.5	22.6	85.0	8.5	1.0	7.0	1.2	0.8	51.7	35.2	HIV–	HIV–	HIV–	HIV–
1204	Male	SECH	63.4	36.1	15.9	76.4	1.7	65.9	4.1	1.3	20.8	21.8	HIV+	HIV+	HIV+	Not cleared
1205	Male	SECH	72.4	25.5	14.6	82.9	0.5	76.2	5.0	2.0	26.0	30.0	HIV–	HIV–	HIV–	HIV–
1206	Male	SECH	73.0	27.0	33.7	57.1	1.3	47.9	6.1	0.0	32.0	52.0	HIV–	HIV–	HIV–	HIV–
1207	Male	SECH	86.0	13.6	36.3	55.9	1.1	48.1	5.8	2.9	34.9	39.0	HIV+	HIV+	HIV+	Not cleared
1208	Male	SECH	79.0	20.2	15.0	78.7	0.7	63.9	9.8	2.3	31.8	36.3	HIV–	HIV–	HIV–	HIV–
1209	Male	SECH	88.7	11.3	40.9	51.9	3.7	48.0	2.6	3.9	34.8	48.3	HIV+	HIV+	HIV+	Not cleared
1210	Female	SECH	60.2	37.7	37.9	56.4	0.9	42.8	11.9	1.8	38.7	45.8	HIV–	HIV–	HIV–	HIV–
1211	Female	SECH	72.5	25.7	37.0	55.3	1.3	44.6	9.6	3.8	44.3	42.4	HIV–	HIV–	HIV–	HIV–
1212	Female	SECH	41.8	56.4	50.2	44.6	1.3	33.5	4.5	4.5	35.8	36.9	HIV+	HIV+	HIV+	Not cleared
1213	Female	SECH	41.6	42.0	22.6	72.7	1.6	50.0	8.4	4.4	29.4	35.2	HIV+	HIV+	HIV+	Not cleared
1214	Female	SECH	30.0	62.8	52.9	42.4	1.2	37.1	4.0	5.2	36.1	37.7	HIV+	HIV+	HIV+	Not cleared
1215	Female	SECH	57.1	41.5	50.4	42.6	2.2	35.4	4.3	4.9	31.4	33.8	HIV+	HIV+	HIV+	Not cleared
1216	Female	SECH	64.1	35.0	51.3	42.3	1.5	27.6	12.4	9.0	37.3	34.9	N/A	HIV-	N/A	HIV–
1217	Male	pART	61.4	38.6	1.6	97.8	0.2	86.4	8.9	4.3	6.4	5.0	HIV+	HIV+	HIV+	Not cleared
1218	Female	pART	78.9	21.1	42.7	55.3	2.0	30.1	25.2	4.6	37.9	46.5	HIV+	HIV+	HIV+	Not cleared
1219	Female	pART	52.9	34.7	53.2	39.5	1.4	24.7	13.3	6.6	39.6	38.7	HIV+	HIV+	HIV+	Not cleared
1220	Female	pART	77.9	22.0	33.0	58.9	1.3	26.4	32.5	3.9	36.9	46.0	HIV+	HIV+	HIV+	Not cleared
1221	Female	pART	44.5	49.5	21.7	72.3	1.0	59.4	7.7	1.4	32.2	34.6	HIV+	HIV+	HIV+	Not cleared
1222	Male	pART	75.2	21.5	58.5	29.1	3.0	23.4	5.0	5.9	29.4	35.3	N/A	HIV+	N/A	Not cleared
		Average	64.0	33.0	41.5	52.1	1.4	40.8	8.6	3.5	34.9	36.3				
		SD	15.6	13.3	22.0	23.1	0.8	21.1	7.5	2.2	9.9	9.8				

Supplementary Table 2. Testing of HIV-1 infections and treatment using Hu-HSC mice.

Mouse ID	Treatment	% mCD45	% hCD45	hCD45+				%hCD4+ T	HIV-1 detection		
				%CD3	%B cell	%CD4	%CD8		Withdrawl (RT-PCR)	TZA	HIV-1 clearance
1001	SECH	41.9	55.9	27.2	60.9	20.8	5.4	11.6	HIV+	HIV+	Not cleared
1002	SECH	16.9	82.1	59.4	22.9	42.2	19.4	34.7	HIV+	HIV+	Not cleared
1003	SECH	8.9	87.4	79.0	2.2	63.9	19.7	55.8	HIV+	HIV+	Not cleared
1004	SECH	59.6	39.4	13.7	80.0	86.8	10.6	34.2	HIV–	HIV–	HIV–
1005	SECH	11.0	85.6	79.9	10.2	71.7	10.0	61.4	HIV–	HIV–	HIV–
1006	SECH	58.8	36.3	45.7	46.6	86.3	10.9	31.3	HIV–	HIV–	HIV–
1007	SECH	20.0	79.0	55.8	19.1	50.7	8.0	40.1	HIV+	HIV+	Not cleared
1008	SECH	29.5	65.8	74.9	12.0	67.4	8.9	44.3	HIV+	HIV+	Not cleared
1009	SECH	77.7	18.5	57.2	15.8	45.4	16.4	8.4	HIV–	HIV–	HIV–
1010	SECH	21.1	77.9	51.8	20.0	43.5	11.8	33.9	HIV+	HIV+	Not cleared
1011	SECH	21.5	75.3	73.8	16.2	67.2	7.4	50.6	HIV+	HIV+	Not cleared
1012	SECH	20.4	78.6	47.0	28.9	43.8	6.8	34.4	HIV–	HIV–	HIV–
1013	ART	77.1	22.9	3.6	92.9	100.0	0.0	22.9	HIV+	HIV+	Not cleared
1014	ART	77.7	21.3	29.1	58.5	80.5	17.3	17.2	HIV+	HIV+	Not cleared
1015	ART	83.9	16.1	1.6	88.2	50.0	40.0	8.1	HIV+	HIV+	Not cleared
1016	ART	99.8	0.2	42.0	46.7	70.9	23.3	0.1	HIV+	HIV+	Not cleared
	Average	45.4	52.7	46.4	38.8	61.9	13.5	30.6			
	SD	30.5	29.9	25.5	29.5	20.9	9.3	18.0			

Supplementary Table 3. Hu-HSC mice used for SECH+JQ-1 treatments for HIV-1 infections.

Mouse ID	SEX	Treatment	% mCD45	% hCD45	hCD45+			hCD45+		hCD45+CD3-CD19-			HIV-1 detection			
					%B cell	%CD3	%NK	%CD4	%CD8	%DC	%M1	%M2	Withdrawl (RT-PCR)	TZA	hmVOA	HIV clearance
1301	Male	SECH	52.3	46.4	75.8	15.9	0.7	12.5	2.6	1.6	46.2	33.7	HIV+	HIV+	N/A	Not cleared
1302	Male	SECH	46.2	53.8	2.0	95.5	0.5	73.4	16.4	1.4	9.5	10.8	HIV+	HIV+	N/A	Not cleared
1303	Male	SECH	63.9	20.1	12.2	85.4	0.4	75.9	6.0	0.0	75.0	0.0	HIV+	HIV+	HIV+	Not cleared
1304	Male	SECH	46.0	52.2	8.6	87.4	0.8	71.8	8.2	11.1	16.7	61.1	HIV+	HIV+	HIV+	Not cleared
1305	Female	SECH	91.1	8.9	22.8	65.8	2.4	51.8	12.3	4.7	33.7	25.0	HIV+	HIV+	N/A	Not cleared
1306	Female	SECH	64.7	32.8	26.8	67.0	0.7	58.9	6.0	1.8	37.4	45.0	HIV–	HIV–	HIV–	HIV–
1307	Female	SECH	76.2	22.0	40.4	54.3	1.4	47.8	5.3	1.5	43.8	43.5	HIV–	HIV–	HIV–	HIV–
1308	Female	SECH	61.8	34.5	35.6	56.9	1.1	41.8	13.6	4.0	42.5	43.1	HIV+	HIV+	HIV+	Not cleared
1309	Female	SECH	41.6	10.6	72.9	17.8	2.1	6.2	9.7	5.9	39.2	35.8	HIV–	HIV–	HIV–	HIV–
1310	Female	SECH	38.8	59.0	20.8	73.9	1.0	57.9	10.5	1.4	30.6	34.3	HIV–	HIV–	HIV–	HIV–
1311	Female	SECH + JQ-1	22.4	76.3	1.22	97.5	76.2	76.3	13.1	0.55	88	10.1	HIV–	HIV–	HIV–	HIV–
1312	Female	SECH + JQ-1	21.8	76.9	0.77	97.7	76.9	73.5	17.2	1.25	87.3	9.63	HIV–	HIV–	HIV–	HIV–
1313	Female	SECH + JQ-1	87.5	10.5	0.36	4.33	10.4	2.5	1.1	0	96.2	1.92	HIV–	HIV–	HIV–	HIV–
1314	Female	SECH + JQ-1	66	33.1	19	72.7	33.1	50.3	16.9	37.5	0	12.5	HIV–	HIV–	HIV–	HIV–
1315	Female	SECH + JQ-1	68.5	30.9	10.9	82.8	30.9	63.9	13.0	0	25	65.6	HIV–	HIV–	HIV–	HIV–
1316	Female	SECH + JQ-1	71	27.7	0.072	95.4	27.4	57.1	29.1	2.05	69.3	20.1	HIV–	HIV–	HIV–	HIV–
1317	Female	SECH + JQ-1	96.5	1.49	11.2	66.4	0.73	31.9	12.9	1.03	62.3	23.3	HIV+	HIV+	HIV+	Not cleared
1318	Male	SECH + JQ-1	61.6	36.6	12.4	82.1	36.6	67.4	9.9	13.3	20.5	97.9	HIV+	HIV+	HIV+	Not cleared
1319	Male	SECH + JQ-1	98.6	0.99	62.5	20	0.99	12.5	5.0	0	66.7	100	HIV–	HIV–	HIV–	HIV–
1320	Male	SECH + JQ-1	23.7	75	0	99.5	75	76.1	20.1	33.3	27.7	78.8	HIV–	HIV–	HIV–	HIV–
1321	Male	SECH + JQ-1	94.2	4.57	70.2	1.65	4.57	0.8	0.8	7.69	34.5	100	HIV–	HIV–	HIV–	HIV–
1322	Male	SECH + JQ-1	29.2	69.6	1.91	96.1	69.6	77.5	14.3	16.7	19.7	88.9	HIV+	HIV+	HIV+	Not cleared
1323	Male	SECH + JQ-1	96.9	2.33	76.2	2.5	2.33	1.3	0.0	40	50	98.4	HIV–	HIV–	HIV–	HIV–
1324	Male	pART	39.5	60.5	23.5	71.3	1.0	64.2	3.9	3.2	28.2	35.9	HIV+	HIV+	N/A	Not cleared
1325	Female	pART	60.3	39.6	36.9	62.5	3.5	40.6	21.9	1.0	35.5	51.2	HIV+	HIV+	N/A	Not cleared
1326	Female	pART	83.5	15.2	37.8	55.6	0.9	47.3	6.7	6.6	28.7	27.7	HIV+	HIV+	N/A	Not cleared
1327	Female	pART	37.6	60.1	70.5	24.8	1.2	16.9	6.5	2.6	38.3	38.7	HIV+	HIV+	N/A	Not cleared
		Average	60.8	35.6	27.9	61.2	17.1	46.6	10.5	7.4	42.7	44.2				
		SD	24.4	24.7	26.7	32.6	26.7	26.6	7.0	11.5	24.5	31.8				
