# Supplementary material for: Clearance of HIV infection by selective elimination of host cells capable of producing HIV
Source: Nat Commun. 2020 Aug 13;11:4051. doi: 10.1038/s41467-020-17753-w (PMC7426846; doi:10.1038/s41467-020-17753-w)
Supplement: Supplementary file 2 — Reporting Summary [file 41467_2020_17753_MOESM2_ESM.pdf]

nature research

Corresponding author(s): Jin Wang

Last updated by author(s): July 12, 2020

Reporting Summary

Nature Research wishes to improve the reproducibility of the work that we publish. This form provides structure for consistency and transparency in reporting. For further information on Nature Research policies, see Authors & Referees and the Editorial Policy Checklist .

Statistics

For all statistical analyses, confirm that the following items are present in the figure legend, table legend, main text, or Methods section.

n/a	Confirmed	
[ ]	[x]	The exact sample size (*n*) for each experimental group/condition, given as a discrete number and unit of measurement
[ ]	[x]	A statement on whether measurements were taken from distinct samples or whether the same sample was measured repeatedly
[ ]	[x]	The statistical test(s) used AND whether they are one- or two-sided<br>*Only common tests should be described solely by name; describe more complex techniques in the Methods section.*
[x]	[ ]	A description of all covariates tested
[x]	[ ]	A description of any assumptions or corrections, such as tests of normality and adjustment for multiple comparisons
[ ]	[x]	A full description of the statistical parameters including central tendency (e.g. means) or other basic estimates (e.g. regression coefficient) AND variation (e.g. standard deviation) or associated estimates of uncertainty (e.g. confidence intervals)
[ ]	[x]	For null hypothesis testing, the test statistic (e.g. *F*, *t*, *r*) with confidence intervals, effect sizes, degrees of freedom and *P* value noted<br>*Give P values as exact values whenever suitable.*
[x]	[ ]	For Bayesian analysis, information on the choice of priors and Markov chain Monte Carlo settings
[x]	[ ]	For hierarchical and complex designs, identification of the appropriate level for tests and full reporting of outcomes
[x]	[ ]	Estimates of effect sizes (e.g. Cohen's *d*, Pearson's *r*), indicating how they were calculated

Our web collection on statistics for biologists contains articles on many of the points above.

Software and code

Policy information about availability of computer code

Data collection	BD FACSDiva™ software (version 9.0, BD Biosciences), Applied Biosystems QuantStudio 5 Real-Time PCR System (Applied Biosystems, Thermo Fisher Scientific)
Data analysis	GraphPad Prism 8 (version 8.3.1), QuantStudio™ Design and Analysis Software (version 1.4), FlowJo (version 10.5.3)

For manuscripts utilizing custom algorithms or software that are central to the research but not yet described in published literature, software must be made available to editors/reviewers. We strongly encourage code deposition in a community repository (e.g. GitHub). See the Nature Research guidelines for submitting code & software for further information.

Data

Policy information about availability of data

All manuscripts must include a data availability statement. This statement should provide the following information, where applicable:

- Accession codes, unique identifiers, or web links for publicly available datasets
- A list of figures that have associated raw data
- A description of any restrictions on data availability

The data generated during this study are included in the paper (and its supplementary information files) or available from the corresponding author upon reasonable request.

Field-specific reporting

Please select the one below that is the best fit for your research. If you are not sure, read the appropriate sections before making your selection.

Life sciences study design

All studies must disclose on these points even when the disclosure is negative.

Sample size	Sample sizes were determined based on pilot experiments and related literatures. Considering the limitation of humanized mouse model, such as GVH (graft versus host), the potential toxicity of drug treatment and the efficacy of treatment, at least 15 mice were employed in the experimental groups, which we have found to be sufficient to produce a statistically reliable results. The number of independent experiments was indicated in each figure legend.
Data exclusions	No data were excluded for data analyses.
Replication	For each experiment, the number of biological independent animals/samples/patients is reported in the figure legend. A total of 8 sets of experiments were performed to establish HIV-1 infections and treatments using HSC-Hu mice, including 3 sets of experiments to test the reconstitution of NSG-SGM3 mice with human CD34+ stem cells and the establishment of HIV-1 infections in these mice, 2 sets of experiments to determine the dose of drugs that could be safely used in C57BL/6 mice and NSG- SGM3-derived HSC-Hu mice. Findings from in vitro cell culture studies were successfully replicated at least 3 independent experiments.
Randomization	Age- and sex-matched animals were randomly assigned to the experimental groups.
Blinding	The investigators were not blinded to experimental group assignments since they need to administrate specific ART or SECH treatments and assess the mice daily according to the regimen procedure.

Reporting for specific materials, systems and methods

We require information from authors about some types of materials, experimental systems and methods used in many studies. Here, indicate whether each material, system or method listed is relevant to your study. If you are not sure if a list item applies to your research, read the appropriate section before selecting a response.

Materials & experimental systems

n/a	Involved in the study	
☐	☒	Antibodies
☐	☒	Eukaryotic cell lines
☒	☐	Palaeontology
☐	☒	Animals and other organisms
☐	☒	Human research participants
☒	☐	Clinical data

Methods

n/a	Involved in the study	
☒	☐	ChIP-seq
☐	☒	Flow cytometry
☒	☐	MRI-based neuroimaging

Antibodies

Antibodies used

Immunofluorescence staining: rabbit anti-human LC3A/B antibody (1:100, 12741s, Cell Signaling Technology), Alexa Fluor 594 donkey anti-rabbit IgG (1:5000, A21207, Thermo Fisher Scientific).

WB: Mouse anti-human caspase-9 (1:1,000, M054-3, Medical & Biological Laboratories); Mouse anti-human caspase-7 (1:1,000, M053-3, Medical & Biological Laboratories); Rabbit anti-human Atg-7 (1:1,000, 2631S, Cell Signaling Technology); Rabbit anti-human cleaved caspase-9 (1:1,000, 52873s, Cell Signaling Technology); Rabbit anti-human caspase-3 (1:1,000, 9665s, Cell Signaling Technology); Rabbit anti-human cleaved caspase-3 (1:1,000, 9501s, Cell Signaling Technology); Rabbit anti- human caspase-6 (1:1,000, 9762s, Cell Signaling Technology); Rabbit anti-human Mcl-1 (1:1,000, 5453s, Cell Signaling Technology); Rabbit anti-human Bcl-2 (1:1,000, 4223s, Cell Signaling Technology); Rabbit anti-human Bcl-xL (1:1,000, 2762s, Cell Signaling Technology); Rabbit anti-human Bak (1:1,000, 12105s, Cell Signaling Technology); Rabbit anti-human Bax (1:1,000, 2772s, Cell Signaling Technology); mouse antibody to human β-Actin (1:50,000, sc-47778, Santa Cruz Biotechnology); Goat Anti-Mouse IgG1-HRP (1:50,000, 1070-05, Southern Biotech); Goat Anti-Rabbit IgG-HRP (1:50,000, ab6721, abcam), Rabbit anti-human LC3 (1:1000, 4108s) from Cell Signaling Technology.

Flow cytometry: The following antibodies from Biolegend were used for flow cytometry: Pacific Blue-anti-mouse CD45 (1:100, 103126, clone 30-F11,) APC-anti-human CD45 (1:100, 304012, clone HI30), Pacific Blue-anti-human CD19 (1:100, 302232, clone HIB19), PE-anti-human CD4 (1:100, 317414, clone OKT4), APC/Fire-750-anti-human CD8 (1:100, 34474, clone SK1), FITC-anti-human CD56 (1:100, 392413, clone QA17A16), Pacific Blue-anti-human CD3 (1:100, 300329, clone HIT3a), PE/Cy7-anti-HLA-HLA-DR, DP, DQ (1:100, 361708, clone Tü39), PerCP/Cy5.5-anti-human CD11b (1:100, 301327, clone ICRF44), APC/Fire-750-anti-human CD163 (1:100, 333633, clone GHI/61), PE-anti-human CD123 (1:100, 306005, clone 6H6), Alexa Fluor 488 anti-human CD11c (1:100, 301618, clone 3.9), PerCP/Cy5.5-anti-human CD3 (1:100, 300328, clone HIT3a), PerCP/Cy5.5 anti-human CD123 (1:100, 306016, clone 6H6), FITC-anti-mouse CD45 (1:100, 103108, clone 30-F11), PE/Cy7 anti-human CD197 (CCR7) (1:100, 353226, clone G043H7), PE-anti-human CD45RO (1:100, 304244, clone UCHL1), FITC-anti-human CD45RA (1:100, 304148, clone HI100) and Alexa Fluor 488-anti-CD68 (1:30, 333812, clone Y1/82A), PerCP/Cy5.5-anti-human CD19 (1:100, 302230, clone HIB19), PE-anti-human CD27 (1:100, 356406, clone M-T271) and Pacific Blue-anti-human IgD (1:100, 348224, clone IA6-2). PE-anti-human CD3 (1:100, 556612, clone SP34) and V50-anti-human CD4 (1:100, 560345, clone RPA-T4) were from BD Biosciences.

	PE-conjugated anti-p24 (1:30, 6604667, clone KC57) was from Beckman Coulter.
Validation	The validation for all antibodies for Western blot and flow cytometry to detect intracellular proteins and cell surface makers have been validated by the suppliers and published on their web pages. These antibodies for flow cytometry were further tested by staining cells with known makers and confirmed by flow cytometry before analyzing experimental samples. Human PBMCs with or without infection by HIV-1 were used as positive and negative controls for staining with PE-anti-HIV-1 p24 to confirm the specificity in the detection of HIV-1 p24 (Supplementary Figure 6a).

Eukaryotic cell lines

Policy information about cell lines

Cell line source(s)	TZM-bl Cell line was obtained from the NIH AIDS Reagent Program (Cat# 8129).
Authentication	TZM-bl Cells were further confirmed by flow cytometry to determine the expression of CD4, CXCR4 and CCR5. The cell line was further tested using HIV-1 standards with known titers to confirm its validity in quantitatively detecting HIV-1.
Mycoplasma contamination	TZM-bl cells was obtained from NIH AIDS Reagent Program and tested to be negative for mycoplasma.
Commonly misidentified lines (See ICLAC register)	No commonly misidentified lines were used.

Animals and other organisms

Policy information about studies involving animals; ARRIVE guidelines recommended for reporting animal research

Laboratory animals	NSG-SGM3 mice were purchased from the Jackson Laboratory. Within 3 days after birth from the mating of NSG-SGM3 mice, newborn male and female mice were implanted intrahepatically with CD34+ human stem cells (AllCells) to generate a humanized mouse model. During treatments, mice were monitored daily for body weight, food consumption, activity and any other discomfort signs related to treatment. At the end of experiment, histological analysis by H&E staining was carried out in the major vital organs (brain, liver, lung and kidney).
Wild animals	No wild animals were used in this study.
Field-collected samples	No field-collected samples were used in this study.
Ethics oversight	Experiments were performed according to federal and institutional guideline, and with the approval of the Institutional Animal Care and Use Committee of the Houston Methodist Research Institute.

Note that full information on the approval of the study protocol must also be provided in the manuscript.

Human research participants

Policy information about studies involving human research participants

Population characteristics	Buffy coats of anonymous healthy donors were purchased from the Gulf Coast Blood Center. The investigators were blinds to any covariate.<br><br>HIV-1+ patients of all age, gender and ethnic background were included. Ten ART-naive patients who were HIV-1+ but never received ART treatments were recruited from The University of Texas Health Science Center at Houston. Ten ART-experience samples from the outpatients undergoing ART treatments were collected at the Houston Methodist Hospital, Houston, Texas.
Recruitment	ART-naive patients were recruited with written informed consents and the approval of the Institutional Review Boards of University of Texas Health Science Center at Houston and Houston Methodist Research Institute. Experiments with ART-naïve patients were performed according to federal and institutional guidelines<br><br>For ART-experienced patients, specimens of ART-treated, HIV-1+ patients from Houston Methodist Hospital Research Institute (HMRI) Biorepository were included. Experiments with de-identified samples from ART-treated patients were performed according to federal and institutional guidelines with the approval of the Institutional Review Board of the Houston Methodist Research Institute. For ART experienced patients, the informed consents for the de-identified biospecimens in Figure 7 from HMRI Biorepository were waived according to the federal regulation 45 CFR 46.116.
Ethics oversight	Samples used in this study were de-identified and assigned with unique identification number before provided to the laboratory for research. This number is used to label the samples that are stored and/or distributed, and also is used to for sample information and/or related clinical data. The identification system is password–protected and is only accessible by the PI and assigned research staff for the study. The protocols for this study were approved and oversaw by The University of Texas at Houston and the Houston Methodist Hospital Research Institute.

Note that full information on the approval of the study protocol must also be provided in the manuscript.

Flow Cytometry

Plots

Confirm that:

- [x] The axis labels state the marker and fluorochrome used (e.g. CD4-FITC).
- [x] The axis scales are clearly visible. Include numbers along axes only for bottom left plot of group (a 'group' is an analysis of identical markers).
- [x] All plots are contour plots with outliers or pseudocolor plots.
- [x] A numerical value for number of cells or percentage (with statistics) is provided.

Methodology

Sample preparation	CD4+ T cells were purified with anti-CD4 MACS beads from peripheral blood mononuclear cells (PBMCs) of anonymous healthy donors and stained with corresponding antibodies on ice for 30 min. Then, CD3+CD4+CD45RA+CD45RO-CCR7+ naïve T cells, CD3 +CD4+CD45RA–CD45RO+CCR7+ central memory T cells (CMT) and CD3+CD4+CD45RA–CD45RO+CCR7–effector memory T cells (EMT) were sorted using a BD FACSAria flow cytometer (BD Bioscience).<br><br>Spleen cells, bone marrow cells and PBMCs from Hu-HSC mice were isolated and red blood cells were lysed with ammonium chloride lysing buffer (0.15 M NH_4Cl, 10 mM $KHCO_3$, 0.1 mM EDTA). The cells then stained with indicated antibodies on ice for 30 min and analyzed using a BD LSR II flow cytometer.<br><br>To detect HIV-1 p24, spleen cells from Hu-HSC mice were stimulated with 5 μg/ml PHA (Sigma) and 6 ng/ml IL-2 (Biolegend). The cells were stained for T cell markers, followed by fixation and permeabilization using the Cytofix/Cytoperm buffer (BD Bioscience) and intracellular staining with PE-conjugated anti-p24 (1:30, 6604667, clone KC57, Beckman Coulter). The cells were analyzed by flow cytometry using a BD LSR II flow cytometer (BD Bioscience)
Instrument	BD LSR II (BD), BD FACSAria (BD)
Software	BD FACSDiva™ software, FlowJo 10.5.3
Cell population abundance	Cell population abundance was indicated in flow cytometry plots.
Gating strategy	Gating and purities of sorted naive T cells, CMT and EMT were shown in Extended Data Figures. Gating and analyses of human immune cells in humanized mice are shown in Extended Data Figures. Human PBMCs with or without HIV-1 infection were used as positive or negative controls to determine the gating strategy for HIV-1 p24 staining as shown in Extended Data Figures.

- [x] Tick this box to confirm that a figure exemplifying the gating strategy is provided in the Supplementary Information.
